# RNF5/185 deficiency reveals a distinct compensatory role for AMFR in mutant CFTR ERAD

**DOI:** 10.1016/j.isci.2026.118195

**Published:** 2026-11-10

**Authors:** Uta Nakayamada, Aika Fujii, Saori Kato, Yuki Karasaki, Mayuko Nakatani, Momone Yamamoto, Natsuki Manai, Yuka Kamada, Tsukasa Okiyoneda

**Affiliations:** 1 Department of Biomedical Sciences, School of Biological and Environmental Sciences, Kwansei Gakuin University, Sanda, Hyogo 669-1330, Japan

**Keywords:** ERAD, ubiquitin ligase, RNF5, RNF185, AMFR, CFTR

## Abstract

Abnormal proteins in the endoplasmic reticulum (ER) are eliminated by ER-associated degradation (ERAD), a quality-control network governed by multiple ubiquitin E3 ligases. We examined how mammalian ERAD maintains degradation capacity when a major E3 ligase pathway is impaired. Here, we identified AMFR as a compensatory factor for mutant cystic fibrosis transmembrane conductance regulator (CFTR) degradation in cells lacking RNF5 and RNF185 (RNF5/185). RNF5/185 double-knockout cells showed increased AMFR protein abundance and reduced AMFR turnover, while AMFR mRNA exhibited an upward trend. AMFR depletion had a relatively limited effect on CFTR degradation in WT cells but markedly impaired degradation in RNF5/185-deficient cells. By contrast, several other ER-associated E3 ligases, including the AMFR homolog SYVN1, did not show a comparable compensatory effect. These findings indicate that AMFR-dependent ERAD functionally compensates for RNF5/185 deficiency and suggest that mammalian ERAD is an adaptable proteostasis network in which E3 ligase contributions shift with cellular context.

## Highlights

• RNF5/185 deficiency increases AMFR abundance and reduces its turnover
• AMFR contributes more strongly to mutant CFTR ERAD upon RNF5/185 loss
• AMFR depletion reduces CFTR ubiquitination in RNF5/185-deficient cells
• AMFR shows a distinct compensatory role among tested ER-associated E3 ligases

## Graphical abstract

**Figure:**
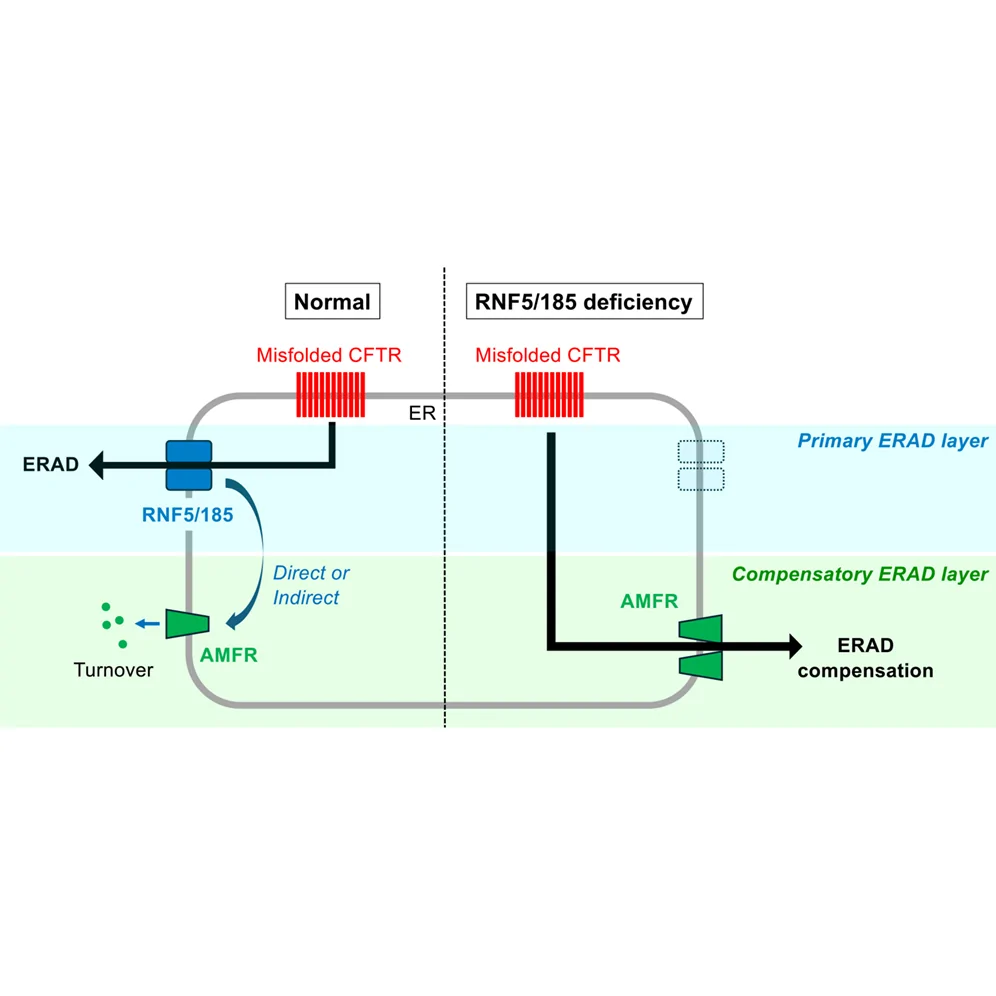

## Teaser

Biological sciences; Biochemistry; Protein; Cell biology

## Introduction

In the endoplasmic reticulum (ER), aberrant proteins, including misfolded and unassembled proteins, are selectively recognized by ER quality control (ERQC) mechanisms and primarily degraded through the ubiquitin (Ub)-proteasome system.^1^^,^^2^ In coordination with molecular chaperones and other accessory factors, Ub ligases facilitate ER-associated degradation (ERAD), a process in which defective proteins are retrotranslocated from the ER to the cytoplasm for degradation by the proteasome.^1^^,^^2^^,^^3^^,^^4^ The specific ERAD pathway used depends on the localization of conformational defects, with different Ub ligases directing proteins to distinct ERAD branches.^2^^,^^5^ The yeast *Saccharomyces cerevisiae* possesses three distinct ERAD branches, each defined by a specific Ub ligase complex: Hrd1, Doa10, and Asi.^2^^,^^5^^,^^6^^,^^7^^,^^8^ These ERAD pathways exhibit substrate specificity and are localized to different regions of the ER, collectively targeting a wide range of misfolded proteins for degradation. The Hrd1 complex plays a key role in eliminating soluble, ER-luminal proteins classified as ERAD-L substrates. Additionally, it participates in the degradation of ERAD-M substrates with structural defects in the membrane.^9^ The Doa10 complex is primarily responsible for degrading ERAD-C substrates, recognizing misfolded cytosolic domains on certain membrane proteins.^10^ Furthermore, Doa10 can detect degradation signals within the ER membrane, facilitating the degradation of ERAD-M substrates, such as unassembled subunits of ER membrane complexes and mislocalized membrane proteins.^11^^,^^12^ The Asi complex, uniquely localized to the inner nuclear membrane (INM), a specialized ER subdomain exposed to the nucleoplasm, is specifically involved in the degradation of mislocalized proteins within the INM.^13^

In mammals, the E3 ligases AMFR (Gp78), Synoviolin 1 (SYVN1), and MARCHF6 (TEB4) share a similar domain organization and topology with their yeast counterparts, but exhibit limited sequence homology. Among them, AMFR and SYVN1 are orthologous to yeast Hrd1, whereas MARCHF6 is the ortholog of yeast Doa10.^2^^,^^8^^,^^14^ Similar to yeast, SYVN1 in mammalian cells plays a crucial role in both the ERAD-L and ERAD-M pathways, facilitating the degradation of aberrant proteins with conformational defects in the ER lumen and intramembrane regions.^3^^,^^4^^,^^15^ AMFR and MARCHF6 are involved in the ERAD-M and ERAD-C pathways, respectively.^16^ The classification of ERAD-M substrates can be complex, as missense mutations or assembly defects in membrane proteins may either disrupt or promote their incorporation into the lipid bilayer.^17^ These mutations may also induce major structural changes, particularly at domain interfaces. In contrast, ERAD-C substrates include large multipass transmembrane proteins with cytosolic domain mutations, such as the ΔF508 mutant of cystic fibrosis transmembrane conductance regulator (CFTR),^18^ improperly integrated tail-anchored proteins,^19^ and cytoplasmic proteins with exposed hydrophobic regions at domain or subunit interfaces.^20^ In addition to the previously mentioned E3 Ub ligases, several ER membrane-bound Ub ligases have been implicated in ERAD in mammalian cells, including RNF5 (RMA1), RNF185, RNF139 (TRC8), RNF145, RNF170, and TMEM129.^2^^,^^8^ Although these ERAD factors are often discussed as components of distinct or partially overlapping branches, the large number of ER-associated Ub ligases complicates the evaluation of their redundancy and cooperation. How the relative contributions of these ligases change when a major ERAD pathway is impaired, and whether specific ligases can functionally compensate under such conditions, remain largely unclear.

The multispanning membrane protein CFTR is a cAMP-regulated chloride (Cl^−^) channel located at the plasma membrane (PM), and its mutations cause cystic fibrosis (CF), one of the most common genetic disorders.^21^ CFTR consists of two membrane-spanning domains (MSD1 and MSD2), two large cytosolic nucleotide-binding domains (NBD1 and NBD2), and a regulatory (R) domain.^21^ The most common CF-associated mutant, ΔF508-CFTR, results from the deletion of a phenylalanine at position 508 in NBD1, causing misfolding in the ER. This misfolded protein undergoes ubiquitination and is subsequently degraded through the ERAD pathway.^22^ ΔF508-CFTR has been classified as an ERAD-C substrate due to its mutation in the cytosolic NBD1.^23^^,^^24^ Multiple Ub E3 ligases have been identified as key regulators of CFTR ERAD. The ER-embedded Ub ligase RNF5 and its paralog RNF185 are thought to mediate the primary CFTR ERAD pathway.^25^^,^^26^^,^^27^ Additionally, cytosolic HERC3 and the chaperone-associated ligase CHIP (STUB1) contribute to alternative ERAD pathways.^27^^,^^28^ AMFR has been reported to function as an E4-like enzyme downstream of the RNF5-mediated ERAD branch, further facilitating CFTR degradation.^29^ Despite the dominant role of RNF5 and RNF185 (RNF5/185) in CFTR degradation, previous studies have shown that some ΔF508-CFTR is still eliminated in their absence.^27^^,^^30^ This residual degradation suggests that alternative ERAD factors can sustain CFTR clearance when RNF5/185 function is lost. However, the factors responsible for this residual activity and the mechanisms that alter their contribution under RNF5/185-deficient conditions remain largely unexplored.

In this study, we used quantitative degradation assays based on luminescent tagging together with combinatorial knockdown (KD)/knockout (KO) approaches to define how the contributions of mammalian ERAD factors change when RNF5/185-dependent degradation is impaired. These analyses uncovered a compensatory role for AMFR: AMFR depletion had a substantially greater effect on mutant CFTR degradation in RNF5/185-deficient cells than in WT cells. We further found that AMFR protein abundance was increased and AMFR turnover was reduced in RNF5/185 DKO cells. Together, these findings identify AMFR as a compensatory ERAD factor whose functional contribution becomes more prominent when RNF5/185-dependent degradation is impaired and provide evidence that the relative contributions of E3 ligase branches can shift within the mammalian ERAD network.

## Results

### AMFR KD markedly increases ΔF508-CFTR levels in RNF5/185 DKO cells

Our previous study, utilizing a HiBiT-based quantitative degradation assay, demonstrated that ΔF508-CFTR is primarily degraded through the RNF5/185-dependent ERAD pathway.^27^ We hypothesized that in RNF5/185 DKO cells, alternative ERAD mechanisms may contribute to the elimination of aberrant proteins that are normally targeted by RNF5/185. To identify candidate genes that may compensate for RNF5/185 function, we compared the mRNA expression levels of ERQC-associated Ub E3 ligases involved in ERAD branches in mammalian cells and other ERAD-related components^2^^,^^8^ between wild-type (WT) and RNF5/185 DKO cells. As a primary screening, RNA sequencing (RNA-seq) analysis revealed that several ERAD-related genes appeared to be upregulated at the mRNA level (Figure 1A). To determine whether these upregulated genes influence ERAD activity, we examined the effects of their KD on ΔF508-CFTR PM expression using ΔF508-CFTR-NanoLuc (Nluc) (Ex), which contains an extracellular Nluc tag, and a cell-impermeable Nluc substrate.^31^ This assay was used because ERAD inhibition can increase ΔF508-CFTR PM levels.^27^ Among the upregulated candidates, including Ub E3 ligases involved in ERAD branches,^2^^,^^8^ only KD of AMFR or UBR4 significantly increased the PM expression of ΔF508-CFTR-Nluc(Ex) in RNF5/185 DKO cells^32^ (Figure 1B). In contrast, KD of AMFR or UBR4 had no significant effect on the PM levels of ΔF508-CFTR-Nluc(Ex) in WT cells (Figure S1). Further western blot analysis revealed that AMFR KD, but not UBR4 KD, led to a substantial increase in both the immature and mature forms of ΔF508-CFTR in RNF5/185 DKO cells (Figure 1C). Based on these findings, we focused on AMFR for further investigation.

**Figure 1:**
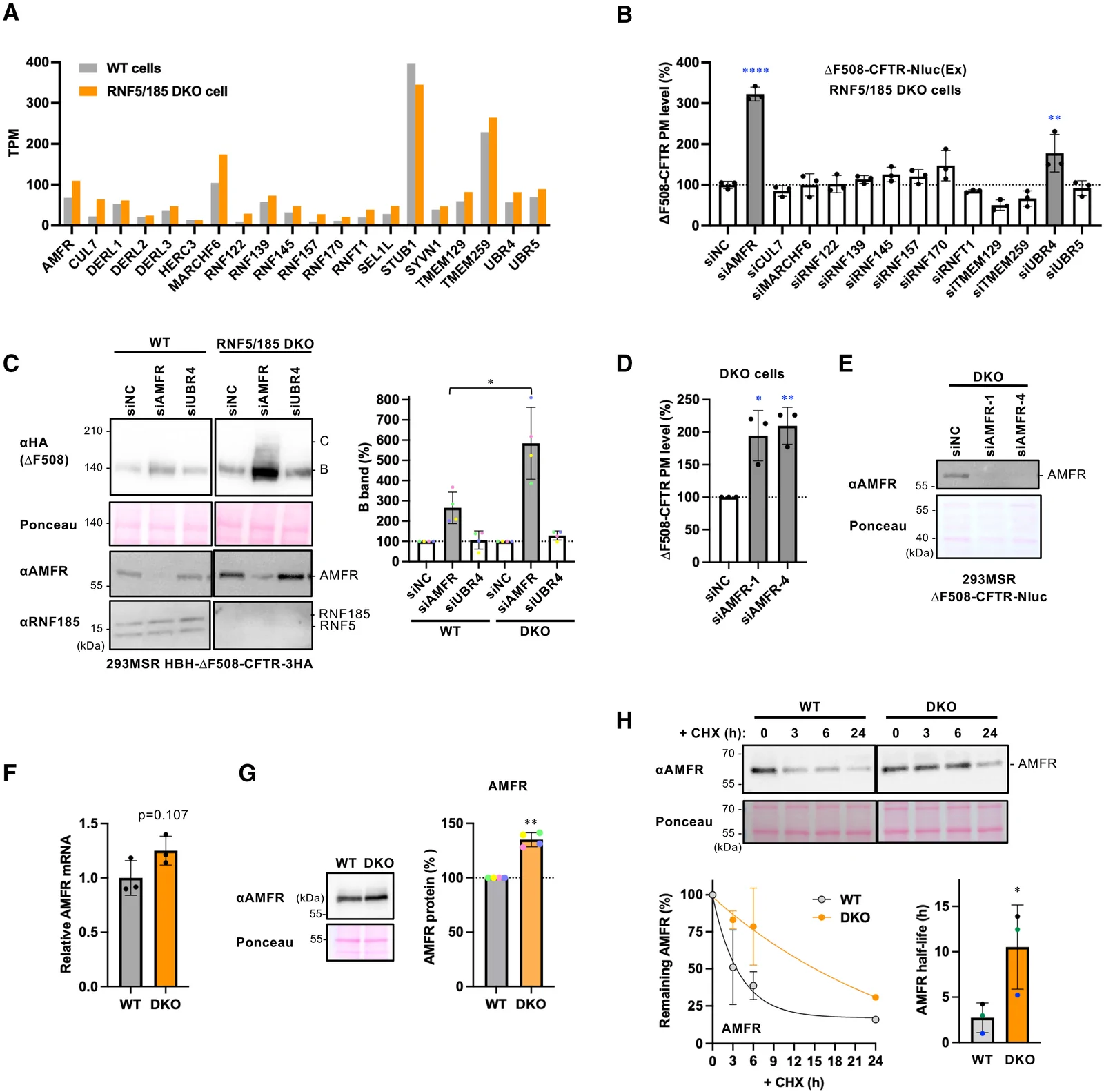
Identification of AMFR as an E3 ligase that limits ΔF508-CFTR expression in RNF5/185 DKO cells (A) RNA-seq analysis highlighting ERAD-related components, including ER-localized E3 ligases, in 293MSR WT and RNF5/185 DKO cells. mRNA expression levels are shown as transcripts per million (TPM; *n* = 1). (B) siRNA screen of E3 ligases that limit cell surface ΔF508-CFTR-Nluc(Ex) in RNF5/185 DKO cells following transfection with 10 nM non-targeting control siRNA (siNC) or pooled siRNAs (*n* = 3). (C) Western blot analysis of HBH-ΔF508-CFTR-3HA in 293MSR WT and RNF5/185 DKO cells transfected with 10 nM pooled siRNAs. Ponceau S staining is shown as a loading control. Band B and Band C represent immature and mature forms of ΔF508-CFTR, respectively. Band B levels were quantified and expressed as a percentage of siNC (right; *n* = 4). Each biological replicate is color-coded. (D) Cell surface expression of ΔF508-CFTR-Nluc(Ex) in RNF5/185 DKO cells transfected with 10 nM individual siRNAs (*n* = 3). (E) Western blot validation of AMFR KD in 293MSR WT and RNF5/185 DKO cells transfected as in (D). (F and G) AMFR mRNA (F; *n* = 3) and protein (G; *n* = 4) levels in 293MSR WT and RNF5/185 DKO cells were measured by qPCR and western blotting, respectively. Each biological replicate is color-coded (G). (H) AMFR turnover in 293MSR WT and RNF5/185 DKO cells was assessed by CHX chase (20 μg/mL) followed by western blotting. Half-life values were determined by curve fitting (*n* = 3). Each biological replicate is color-coded. Ponceau S staining is shown as a loading control. Statistical significance was assessed using one-way ANOVA with Dunnett’s multiple comparisons test (B, D), a two-tailed paired Student’s *t* test (C, G, H), or Welch’s ttests (F). Data are presented as mean ± SD. Data distribution was assumed to be normal but not formally tested. ∗*p* < 0.05, ∗∗*p* < 0.01, ∗∗∗∗*p* < 0.0001.

To further evaluate whether the observed phenotype was reproducibly caused by AMFR depletion, we tested multiple individual siRNAs targeting AMFR using PM CFTR abundance as a quantitative readout. The Nluc assay showed that AMFR KD, using either siAMFR-1 or siAMFR-4, significantly increased the PM level of ΔF508-CFTR in RNF5/185 DKO cells (Figure 1D). Western blot analysis further confirmed that these siRNAs reduced endogenous AMFR protein levels (Figure 1E).

RNA-seq analysis suggested an increase in AMFR mRNA expression, and qPCR analysis showed a similar upward trend in RNF5/185 DKO cells; however, this difference did not reach statistical significance (Figure 1F). In contrast, western blot analysis revealed a statistically significant increase in endogenous AMFR protein abundance in RNF5/185 DKO cells (Figure 1G). To further investigate this discrepancy, we performed cycloheximide (CHX) chase assays to assess AMFR protein turnover. These experiments showed that endogenous AMFR in WT cells was degraded relatively rapidly, with a half-life of approximately 3 h (Figure 1H). In contrast, AMFR degradation was significantly reduced in RNF5/185 DKO cells, with a more than 3-fold increase in its half-life (Figure 1H). These results indicate that increased AMFR protein stability contributes to AMFR accumulation in RNF5/185 DKO cells. However, because AMFR mRNA also showed an upward trend, an additional contribution from transcriptional or post-transcriptional regulation cannot be excluded.

### AMFR makes a greater contribution to the CFTR ERAD in RNF5/185 DKO cells

To quantify the disappearance of fully synthesized ΔF508-CFTR after translation arrest, we used the C-terminal HiBiT degradation assay.^27^^,^^33^ In this assay, HiBiT is fused to the cytosolic C-terminus of CFTR and complements cytosolic LgBiT; following CHX addition, the decline in luminescence reports loss of the C-terminally completed CFTR pool. Thus, the assay provides a sensitive real-time measure of CFTR degradation and is expected to emphasize post-translational degradation of fully synthesized CFTR. The assay has shown strong concordance with conventional western blot-based CHX chase measurements.^27^ Our highly quantitative HiBiT-based degradation assay showed that AMFR KD only modestly reduced ΔF508-CFTR ERAD in 293MSR WT cells (Figure 2A). This limited effect may reflect the dominance of the RNF5/185-dependent pathway under basal conditions, which could mask the contribution of AMFR when RNF5/185 function remains intact. As previously reported,^27^ ERAD of ΔF508-CFTR was markedly reduced in RNF5/185 DKO cells compared with WT cells (Figure 2A). Notably, although AMFR KD had a modest effect on ERAD in WT cells, it robustly inhibited ΔF508-CFTR ERAD in RNF5/185 DKO cells (Figure 2A). Efficient KD of endogenous AMFR protein was confirmed in siRNA-transfected cells (Figure 2B). Similar results were obtained in BEAS-2B airway epithelial cells. AMFR KD had little detectable effect on ΔF508-CFTR-Nluc(CT) degradation in control cells, whereas it significantly reduced ΔF508-CFTR degradation when RNF5 and RNF185 were simultaneously depleted by siRNA (Figures 2C and 2D). However, ΔF508-CFTR degradation was faster in BEAS-2B cells than in 293MSR cells, and the inhibitory effect of combined RNF5/185 and AMFR KD (TKD) was less pronounced than that observed in 293MSR cells (Figure 2C). These observations raise the possibility that airway epithelial cells may have additional degradation mechanisms and/or more stringent ER quality-control capacity to eliminate misfolded CFTR.

**Figure 2:**
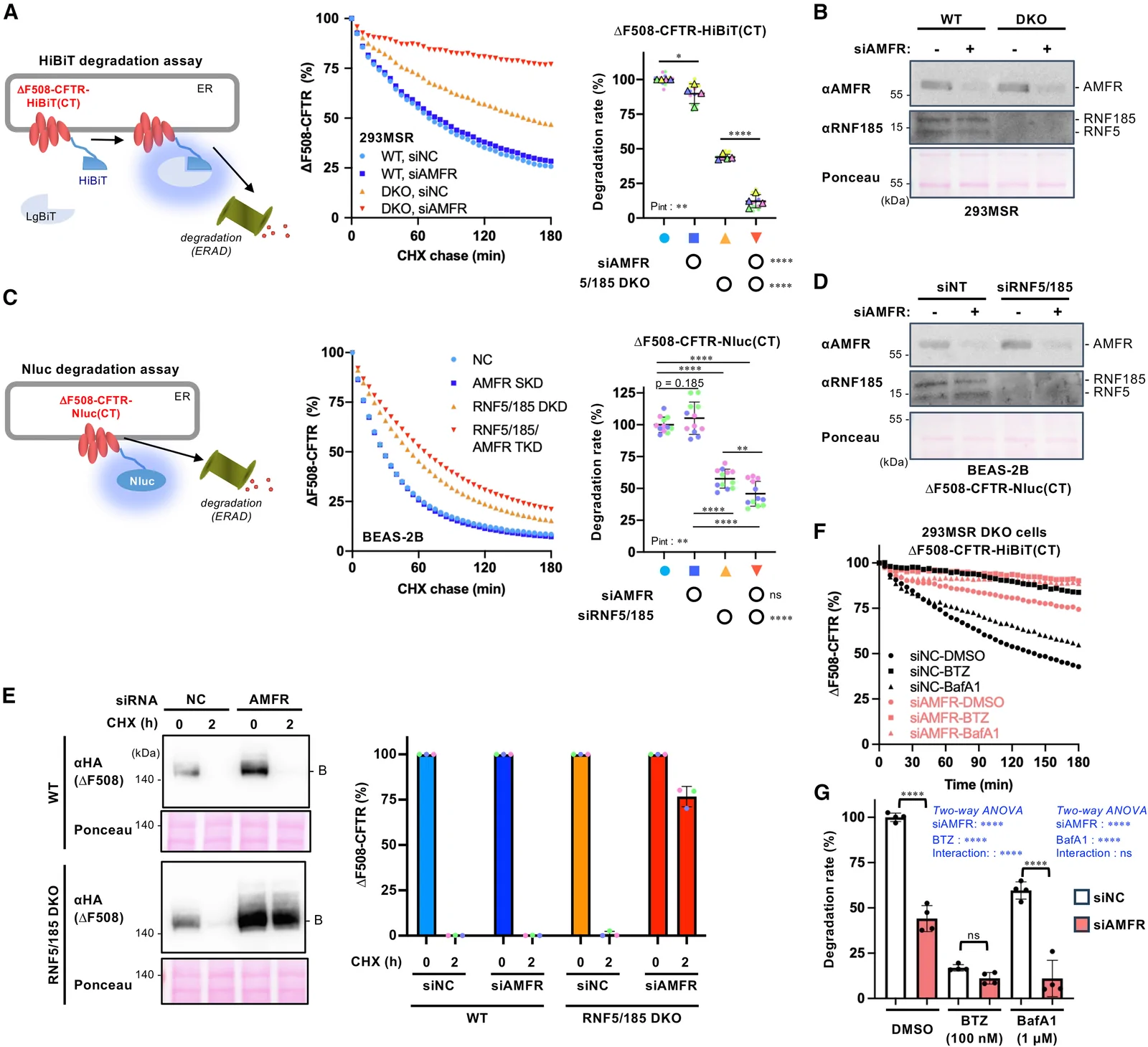
AMFR facilitates the ERAD of ΔF508-CFTR in RNF5/185 DKO cells (A) Kinetic degradation analysis of ΔF508-CFTR-HiBiT(CT) in 293MSR WT and RNF5/185 DKO cells transfected with 10 nM siNC or pooled siAMFR. Luminescence was continuously measured over 180 min in the presence of CHX and normalized to untreated cells. The degradation rate of ΔF508-CFTR-HiBiT(CT) was calculated by fitting the initial degradation phase of each kinetic curve (right, *n* = 4). Each biological replicate (n) is color-coded, with averages from four technical replicates represented as triangles. Statistical significance was assessed using two-way RM ANOVA with Holm-Sidak multiple comparison tests, which revealed significant main effects of AMFR KD and RNF5/185 DKO, as well as a significant statistical interaction between them (P_int_ < 0.05). (B) Western blot confirmation of AMFR KD in 293MSR WT or RNF5/185 DKO cells transfected as in panel A. (C) Kinetic degradation analysis of ΔF508-CFTR-Nluc(CT) in BEAS-2B cells (*n* = 12) transfected with 10 nM pooled siAMFR and/or siRNF5/185 (50 nM pooled siRNF5 + 50 nM pooled siRNF185). The degradation rate of ΔF508-CFTR-Nluc(CT) was calculated as in panel A. Each independent experiment, consisting of four biological replicates (n), is color-coded. Two-way ANOVA with Holm-Sidak multiple comparison tests revealed a significant main effect of RNF5/185 KD, but not AMFR KD, and a significant statistical interaction between AMFR KD and RNF5/185 KD (P_int_ < 0.05). Here, the statistical interaction term tests whether the effect of AMFR KD differs according to RNF5/185 KD status. (D) Western blot confirmation of AMFR and RNF5/185 KD in BEAS-2B cells transfected as in panel (C). (E) Western blot analysis of CHX chase measuring ΔF508-CFTR degradation in 293MSR WT or RNF5/185 DKO HBH-ΔF508-CFTR-3HA cells transfected with 10 nM siNC or pooled siAMFR. Band B (immature ΔF508-CFTR) levels in WT and DKO cells were quantified and expressed as % of T-0 (right, *n* = 3). Each biological replicate (n) is color-coded. (F and G) ΔF508-CFTR-HiBiT(CT) degradation in RNF5/185 DKO cells transfected with 10 nM siNC or siAMFR-1, measured in the presence of 0.1% DMSO, 100 nM bortezomib (BTZ), or 1 μM bafilomycin A1 (BafA1). The degradation rate was calculated by curve fitting of each kinetic degradation curve (G, *n* = 4). Two-way ANOVA was used to test AMFR KD and inhibitor treatment as factors. A significant statistical interaction between AMFR KD and BTZ treatment was detected, indicating that the effect of AMFR KD on the degradation rate depended on BTZ treatment. In contrast, no significant statistical interaction between AMFR KD and BafA1 treatment was detected. Holm-Sidak-adjusted pairwise comparisons are indicated directly in panel (G) to show the relevant group comparisons. Data distribution was assumed to be normal but was not formally tested. Data represent mean ± SD. ∗*p* < 0.05, ∗∗*p* < 0.01, ∗∗∗∗*p* < 0.0001, ns (not significant).

Consistent with the HiBiT-based degradation assays, conventional CHX chase analysis followed by western blotting showed that, after a 2-h chase, AMFR KD had little detectable effect on the elimination of immature ΔF508-CFTR in WT cells, whereas it substantially delayed its elimination in RNF5/185 DKO cells (Figure 2E). These results support the conclusion that the inhibitory effect of AMFR KD reflects impaired CFTR degradation rather than interference with the HiBiT/LgBiT detection system.

Given recent studies indicating that AMFR participates not only in ERAD but also in the ER-phagy pathway,^34^^,^^35^ we examined whether AMFR-dependent degradation in RNF5/185 DKO cells was affected by proteasome inhibition (bortezomib, BTZ) or lysosome inhibition (bafilomycin A1, BafA1). BTZ strongly inhibited ΔF508-CFTR degradation, whereas BafA1 caused a partial reduction in degradation (Figures 2F and 2G), consistent with a predominant contribution of proteasomal degradation and an additional contribution of lysosome-dependent turnover. Two-way ANOVA revealed a significant statistical interaction between AMFR KD and BTZ treatment, indicating that the effect of AMFR KD on CFTR degradation depended on whether proteasomal degradation was inhibited. In contrast, no significant statistical interaction was detected between AMFR KD and BafA1 treatment (Figure 2G). These results are consistent with AMFR contributing primarily to proteasome-dependent CFTR ERAD under these conditions, although they do not exclude an additional contribution of lysosome-dependent pathways to CFTR turnover.

### AMFR contributes to the removal of misfolded CFTR from the ER in WT and RNF5/185 DKO cells

To assess an earlier ERAD step, we used cytosolic LgBiT together with ΔF508-CFTR-HiBiT(Ex), in which the HiBiT tag is positioned in an ER-luminal/extracellular loop of CFTR.^27^ During extraction from the ER membrane, cytosolic accessibility of the luminal HiBiT tag permits complementation with cytosolic LgBiT; MG-132 is used to allow accumulation of the resulting luminescent species. Accordingly, this assay provides an operational readout of cytosolic accessibility associated with ER extraction rather than a direct physical measurement of membrane translocation. Because the internal HiBiT tag is synthesized before the CFTR C-terminus, this assay does not require completion of full-length CFTR synthesis before a tagged species can become detectable and therefore cannot distinguish post-translational from possible co-translational quality-control events. AMFR KD significantly reduced the calculated retrotranslocation-associated rate in 293MSR WT cells (Figure 3A). Consistent with our previous findings,^27^ RNF5/185 DKO cells showed a lower retrotranslocation-associated rate, and AMFR KD further reduced this rate in the DKO background (Figure 3A). Two-way RM ANOVA detected significant main effects of AMFR KD and RNF5/185 DKO but no significant statistical interaction between them, indicating that an AMFR-dependent effect on the retrotranslocation-associated rate was detectable in both genetic backgrounds.

**Figure 3:**
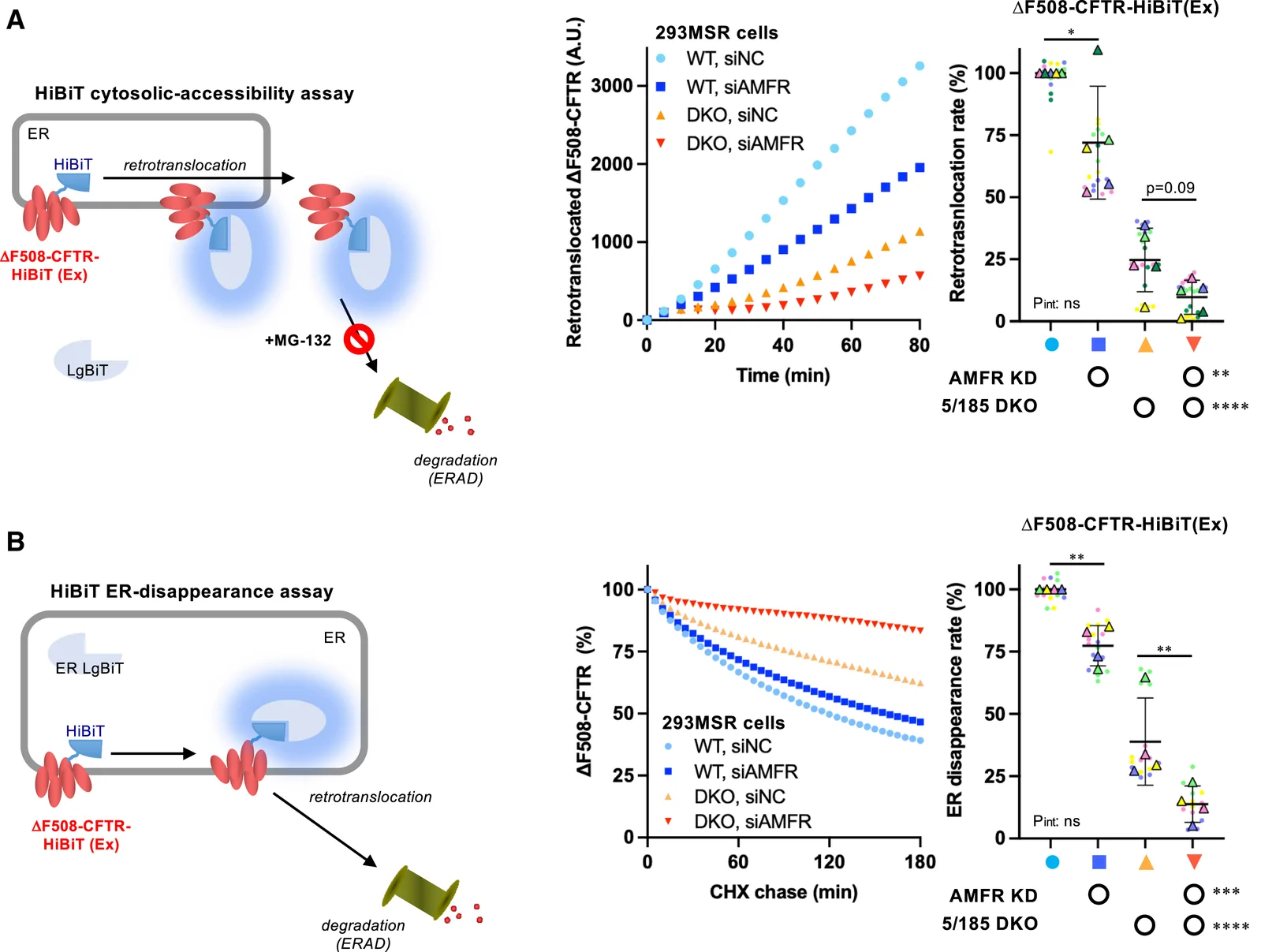
AMFR contributes to the removal of ΔF508-CFTR from the ER in WT and RNF5/185 DKO cells (A) Kinetic analysis using the HiBiT cytosolic-accessibility assay, which provides a retrotranslocation-associated readout of ΔF508-CFTR-HiBiT(Ex) extraction from the ER, in 293MSR WT and RNF5/185 DKO cells transfected with 10 nM siNC or pooled siAMFR. Luminescence was continuously monitored over 80 min in the presence of 10 μM MG-132. The MG-132-dependent increase in complementation between ER-luminal HiBiT and cytosolic LgBiT was used as an operational readout of cytosolic accessibility associated with CFTR extraction, and the retrotranslocation-associated rate was calculated by linear fitting (right, *n* = 5). Two-way RM ANOVA revealed significant main effects of AMFR KD and RNF5/185 DKO, but no significant statistical interaction between them (P_int_ > 0.05). (B) Kinetic ER-disappearance analysis of ΔF508-CFTR-HiBiT(Ex) in 293MSR WT and RNF5/185 DKO cells transfected with 10 nM siNC or pooled siAMFR. Luminescence was continuously monitored over 180 min in the presence of CHX and normalized to untreated cells to represent the remaining ER-lumen-accessible CFTR pool (%). The ER-disappearance rate of ΔF508-CFTR-HiBiT(Ex) was calculated by fitting the kinetic ER-disappearance curve (right, *n* = 4). Two-way RM ANOVA with Holm-Sidak multiple comparison tests revealed significant main effects of AMFR KD and RNF5/185 DKO, but no significant statistical interaction between them (P_int_ > 0.05). Each biological replicate (n) is color-coded. Data distribution was assumed to be normal but was not formally tested. Data represent mean ± SD. ∗*p* < 0.05, ∗∗*p* < 0.01, ∗∗∗*p* < 0.001, ∗∗∗∗*p* < 0.0001, ns (not significant).

To complement the cytosolic-accessibility assay, we used the HiBiT ER-disappearance assay, which combines ER-luminal LgBiT with the same ΔF508-CFTR-HiBiT(Ex) construct.^27^ After CHX addition, loss of luminescence reports disappearance of the HiBiT-tagged CFTR pool that remains accessible to LgBiT within the ER lumen. This loss is consistent with ER extraction followed by degradation but is not specific for retrotranslocation and could also reflect other processes that remove the tagged protein from the ER-luminally accessible pool. AMFR KD reduced the disappearance of ΔF508-CFTR-HiBiT(Ex) from the ER lumen regardless of RNF5/185 status (Figure 3B). Together, these complementary live-cell kinetic assays support a role for AMFR in steps involved in the removal of misfolded CFTR from the ER in both WT and RNF5/185 DKO cells, while neither assay alone establishes a direct retrotranslocation mechanism. Although AMFR KD significantly reduced the retrotranslocation-associated rate in WT cells, its effect on overall CFTR degradation was comparatively limited when RNF5/185-dependent degradation remained intact. By contrast, in RNF5/185 DKO cells, the further reduction in the retrotranslocation-associated rate upon AMFR KD may have a greater functional impact on CFTR ERAD, as the major RNF5/185-dependent pathway is absent.

### AMFR facilitates the ubiquitination of misfolded CFTR in RNF5/185 DKO cells

AMFR has been proposed to function as an E4-like enzyme, facilitating CFTR ubiquitination by extending polyubiquitin (polyUb) chains initiated by the E3 ligase RNF5.^29^ To determine whether AMFR regulates CFTR ubiquitination even in the absence of RNF5/185, we assessed CFTR ubiquitination using multiple approaches, including HBH-based purification under denaturing conditions. The HBH tag, which consists of tandem His_6_ tags separated by a biotinylation sequence, enables efficient isolation of ubiquitinated proteins under denaturing conditions.^36^^,^^37^ Western blotting of isolated HBH-ΔF508-CFTR under denaturing conditions by Neutravidin (NA) pull-down showed that AMFR KD reduced pan-ubiquitination in both WT and RNF5/185 DKO cells (Figures 4A and 4B). Because CFTR ubiquitination levels were low in RNF5/185 DKO cells,^27^ twice the amount of NA pull-down sample was loaded for DKO cells compared with WT cells, as indicated by “2×” in Figure 4A. Additionally, Ub ELISA^36^ using a K48-linked polyUb-specific antibody showed that AMFR KD reduced K48-linked polyubiquitination of CFTR in WT cells (Figure 4C). Although the magnitude of this effect was smaller in RNF5/185 DKO cells, AMFR KD still modestly decreased CFTR K48-linked polyubiquitination (Figure 4C).

**Figure 4:**
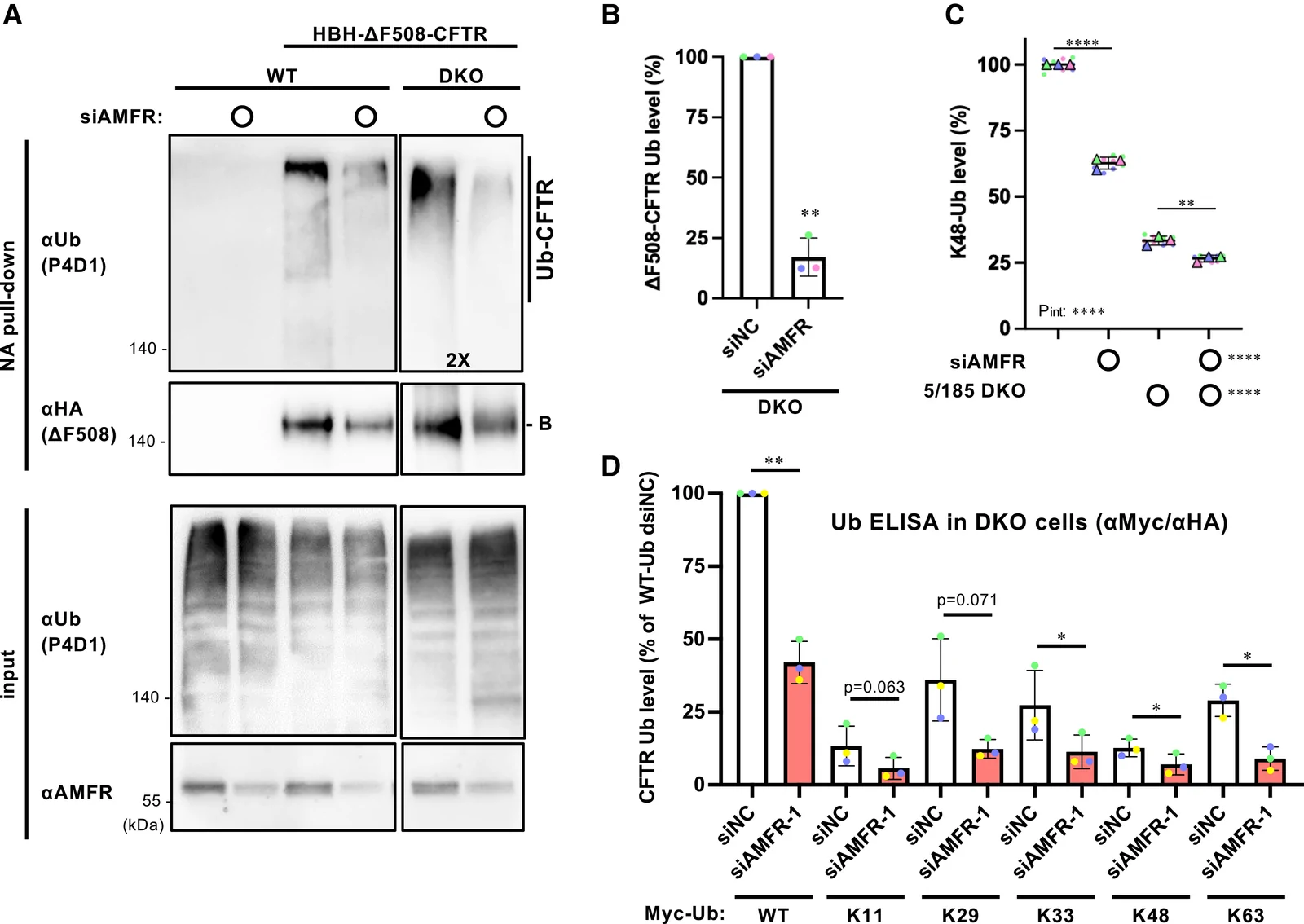
AMFR facilitates the ubiquitination of ΔF508-CFTR in RNF5/185 DKO cells (A and B) Ubiquitination levels of ΔF508-CFTR in HBH-ΔF508-CFTR-3HA 293MSR WT and RNF5/185 DKO cells transfected with 10 nM siNC or pooled siAMFR were measured by NA pull-down under denaturing conditions, followed by western blot analysis. To enhance detection of CFTR ubiquitination in DKO cells, twice the amount of precipitated sample was loaded for DKO cells compared with WT cells, as indicated by 2×. The CFTR ubiquitination level in RNF5/185 DKO cells was quantified by densitometry and normalized to CFTR levels in the precipitates (B). Statistical significance was assessed using a two-tailed paired *t* test (*n* = 3). (C) K48-linked polyubiquitination of ΔF508-CFTR in HBH-ΔF508-CFTR-3HA 293MSR WT and RNF5/185 DKO cells transfected with 10 nM siNT or pooled siAMFR was quantified by Ub ELISA using a K48-linkage-specific polyUb antibody. Cells were treated with 10 μM MG-132 for 3 h at 37°C. The ubiquitination level was normalized to the CFTR amount quantified by ELISA using an anti-HA antibody (*n* = 3). Two-way RM ANOVA with Holm-Sidak multiple comparison tests revealed significant main effects of AMFR KD and RNF5/185 DKO, as well as a significant statistical interaction between them (P_int_ < 0.01). (D) Analysis of various polyUb linkages on ΔF508-CFTR in RNF5/185 DKO HBH-ΔF508-CFTR-3HA cells transfected with Myc-Ub variants and 10 nM siNC or siAMFR-1. Ub ELISA was performed using anti-Myc and anti-HA antibodies (*n* = 3). The Ub levels were expressed as a percentage relative to the siNC and Myc-WT Ub-transfected sample. Each biological replicate (n) is color-coded. Statistical significance was assessed using a two-tailed paired *t* test. Data distribution was assumed to be normal but was not formally tested. Data represent mean ± SD. ∗*p* < 0.05, ∗∗*p* < 0.01, ∗∗∗∗*p* < 0.0001.

Because ERAD substrates can be modified by non-K48 linkages, mixed chains, and branched chains that facilitate proteasomal degradation,^38^ we further investigated the role of AMFR in polyUb chain formation. To this end, we analyzed the effects of AMFR KD on different Ub-chain configurations using ELISA with Myc-Ub mutants, each containing only a single lysine (K) residue. AMFR KD reduced or tended to reduce all tested polyUb chain types on ΔF508-CFTR in RNF5/185 DKO cells, including K29-, K33-, and K63-linked chains (Figure 4D). These results suggest that AMFR contributes to the formation of diverse Ub-chain architectures on CFTR, even in the absence of RNF5/185, thereby supporting its role in compensatory ERAD.

### SYVN1 and other ER-associated E3 ligases do not show a comparable compensatory contribution to CFTR ERAD in RNF5/185 DKO cells

SYVN1 is a mammalian homolog of yeast Hrd1 and shares amino acid sequence homology with AMFR.^39^^,^^40^ However, despite this similarity, SYVN1 and AMFR have distinct cellular functions.^29^^,^^41^ To determine whether SYVN1 also contributes to the compensatory ERAD in RNF5/185 DKO cells, we examined the effect of SYVN1 KD on ΔF508-CFTR degradation. Unlike AMFR KD, SYVN1 KD showed no significant inhibitory effect on ΔF508-CFTR-HiBiT(CT) degradation in either WT or RNF5/185 DKO cells (Figure S2A). Efficient SYVN1 KD using two independent siRNAs was confirmed by western blotting (Figure S2B). Consistent with the degradation assay results, SYVN1 KD had a weaker effect on ΔF508-CFTR protein levels than AMFR KD, especially in RNF5/185 DKO cells (Figure S2B). Furthermore, unlike AMFR KD, SYVN1 KD did not increase the PM expression of ΔF508-CFTR-Nluc(Ex) in RNF5/185 DKO cells (Figure S2C). Ub ELISA showed that SYVN1 KD caused a modest reduction in K48-linked polyubiquitination of ΔF508-CFTR in 293MSR WT cells; however, this reduction was not accompanied by a measurable defect in ΔF508-CFTR degradation (Figure S2D). In RNF5/185 DKO cells, SYVN1 KD did not reduce K48-linked polyubiquitination of CFTR (Figure S2D).

In addition to RNF5/185, several E3 ligases, including CHIP, UBE3C, and HERC3, have been implicated in CFTR ERAD^27^^,^^28^^,^^33^^,^^42^ and are generally thought to function in partially overlapping pathways in CFTR degradation. To determine whether these ligases operate similarly to AMFR, we evaluated the effects of their KD in both WT and RNF5/185 DKO cells. HiBiT-based degradation assays showed that KD of CHIP, UBE3C, or HERC3 caused a modest reduction in ΔF508-CFTR-HiBiT(CT) degradation in WT cells (Figures 5A–5C). Furthermore, in RNF5/185 DKO cells, KD of these E3 ligases also produced only limited inhibition of CFTR degradation and did not recapitulate the robust degradation defect observed upon AMFR KD (Figures 5A–5C). KD of these E3 ligases was confirmed by western blotting or qPCR (Figure 5D).

**Figure 5:**
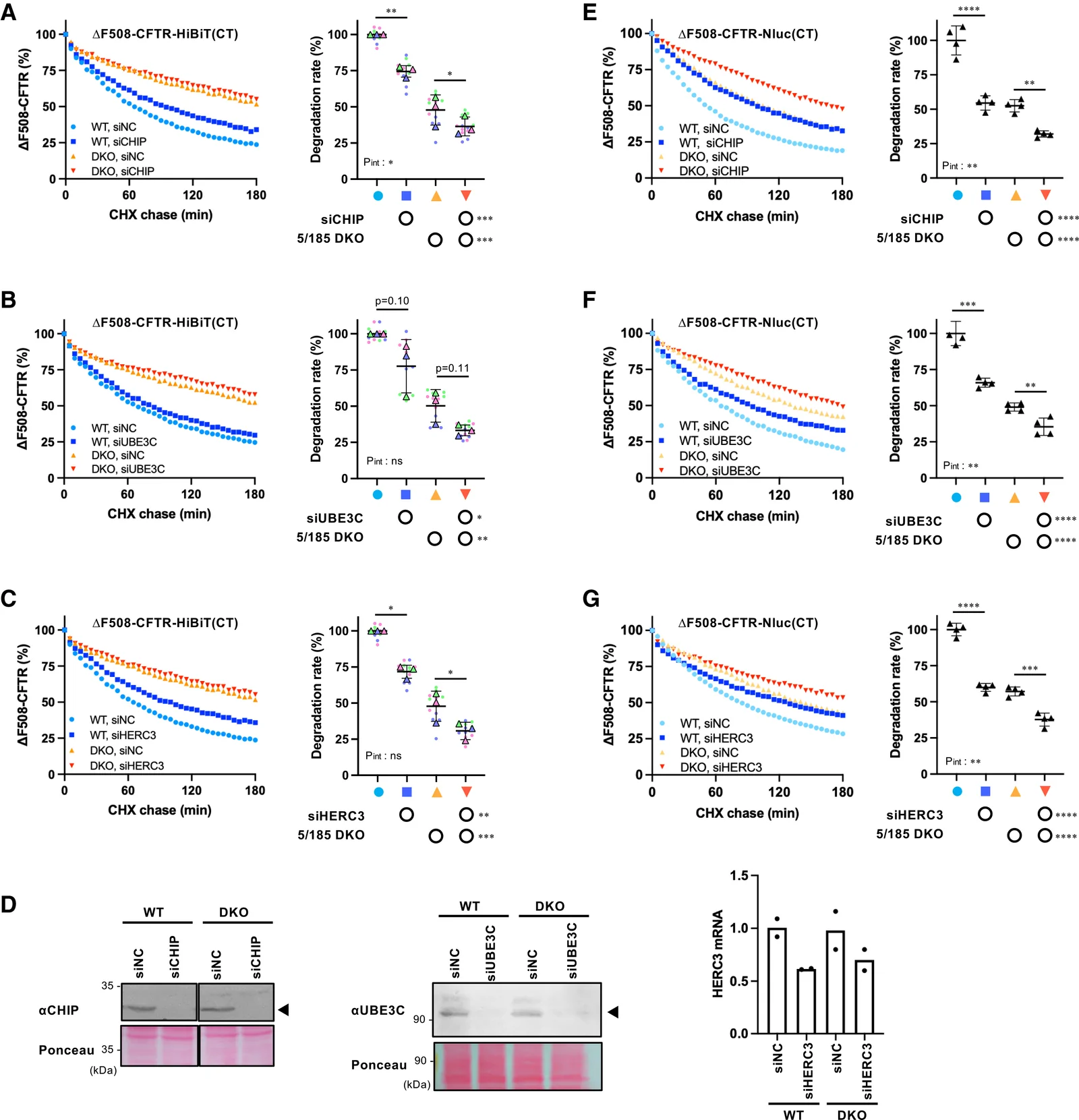
Effects of CHIP, UBE3C, and HERC3 KD on CFTR ERAD in RNF5/185 DKO cells (A–C) Kinetic degradation of ΔF508-CFTR-HiBiT(CT) in 293MSR WT and RNF5/185 DKO cells transfected with the indicated siRNAs was assessed as described in Figure 2A. Degradation rates were determined by fitting the initial phase of the degradation curves (right; *n* = 3). Each biological replicate is color-coded, and the mean of three to four technical replicates is shown as triangles. (D) KD of CHIP, UBE3C, and HERC3 was validated in 293MSR WT and RNF5/185 DKO cells by western blotting and qPCR. (E–G) Kinetic degradation of ΔF508-CFTR-Nluc(CT) in 293MSR WT and RNF5/185 DKO cells transfected with the indicated siRNAs was analyzed as in (A–C). Statistical significance was assessed using two-way repeated-measures (RM) ANOVA (A–C) or two-way ANOVA (E–G) with Holm-Sidak multiple comparison tests. Data are presented as mean ± SD. ∗*p* < 0.05, ∗∗*p* < 0.01, ∗∗∗*p* < 0.001, ∗∗∗∗*p* < 0.0001, ns (not significant).

We next examined the effects of KD of these E3 ligases on ΔF508-CFTR-Nluc(CT) degradation. Consistent with the HiBiT-based degradation assay, KD of CHIP, UBE3C, or HERC3 moderately impaired ΔF508-CFTR-Nluc(CT) degradation in both WT and RNF5/185 DKO cells, although the effects tended to be weaker in DKO cells than in WT cells (Figures 5E–5G). These findings suggest that CHIP, UBE3C, and HERC3 may contribute to CFTR degradation through pathways that function in parallel with RNF5/185 under basal conditions and after RNF5/185 impairment. In contrast, AMFR appears to have a relatively limited role under basal conditions but becomes functionally more important when RNF5/185-dependent degradation is compromised.

In addition to AMFR, other ERAD-associated E3 ligases may be upregulated in RNF5/185 DKO cells and potentially contribute to compensatory CFTR ERAD. To examine this possibility, we tested the effects of KD of several ER-associated E3 ligases in the RNF5/185 DKO background, including RNF170, RNF139, RNF145, TMEM129, RNF122, CUL7, RNFT1, RNF157, RNF213, RNF150, and MARCHF6.^1^^,^^2^^,^^8^^,^^43^^,^^44^^,^^45^^,^^46^^,^^47^^,^^48^^,^^49^^,^^50^ Degradation assays showed that KD of most of these E3 ligases did not produce a clear effect on ΔF508-CFTR-Nluc(CT) degradation in either 293MSR WT or RNF5/185 DKO cells (Figures S3A–S3J). MARCHF6 KD modestly reduced ΔF508-CFTR degradation in RNF5/185 DKO cells; however, this effect appeared to be weaker than that observed upon AMFR KD (Figures S3K and S3L). These results support the idea that AMFR has a distinct compensatory role in CFTR degradation when RNF5/185 function is impaired.

### Substrate selectivity of AMFR-mediated compensatory ERAD

To define the substrate selectivity of AMFR-mediated compensatory ERAD within the ER quality-control system, we examined the effect of AMFR KD on the degradation of various membrane proteins in RNF5/185 DKO cells (Figure 6A). Insig-1 and TCRα have been classified as ERAD-M^16^^,^^51^ and ERAD-Lm^52^ substrates, respectively. ΔY490-ABCB1 (MDR1/P-glycoprotein) is an ERAD-C substrate analogous to ΔF508-CFTR^53^ and is degraded via the RNF5/185 ERAD pathway.^27^ The HiBiT degradation assay showed that AMFR KD significantly inhibited Insig-1 degradation, as reported previously,^51^ whereas it had little detectable effect on TCRα degradation in 293MSR WT cells (Figures 6B and 6C). Consistent with previous studies,^27^ Insig-1 and TCRα degradation was unexpectedly accelerated in RNF5/185 DKO cells, possibly reflecting greater contributions from alternative degradation pathways (Figures 6B and 6C). Notably, AMFR KD had a stronger inhibitory effect on Insig-1 and TCRα degradation in RNF5/185 DKO cells than in WT cells, resulting in substantial stabilization of these substrates (Figures 6B and 6C). In contrast, AMFR KD significantly reduced ΔY490-ABCB1 degradation in WT cells, but this effect was less pronounced in RNF5/185 DKO cells (Figure 6D).

**Figure 6:**
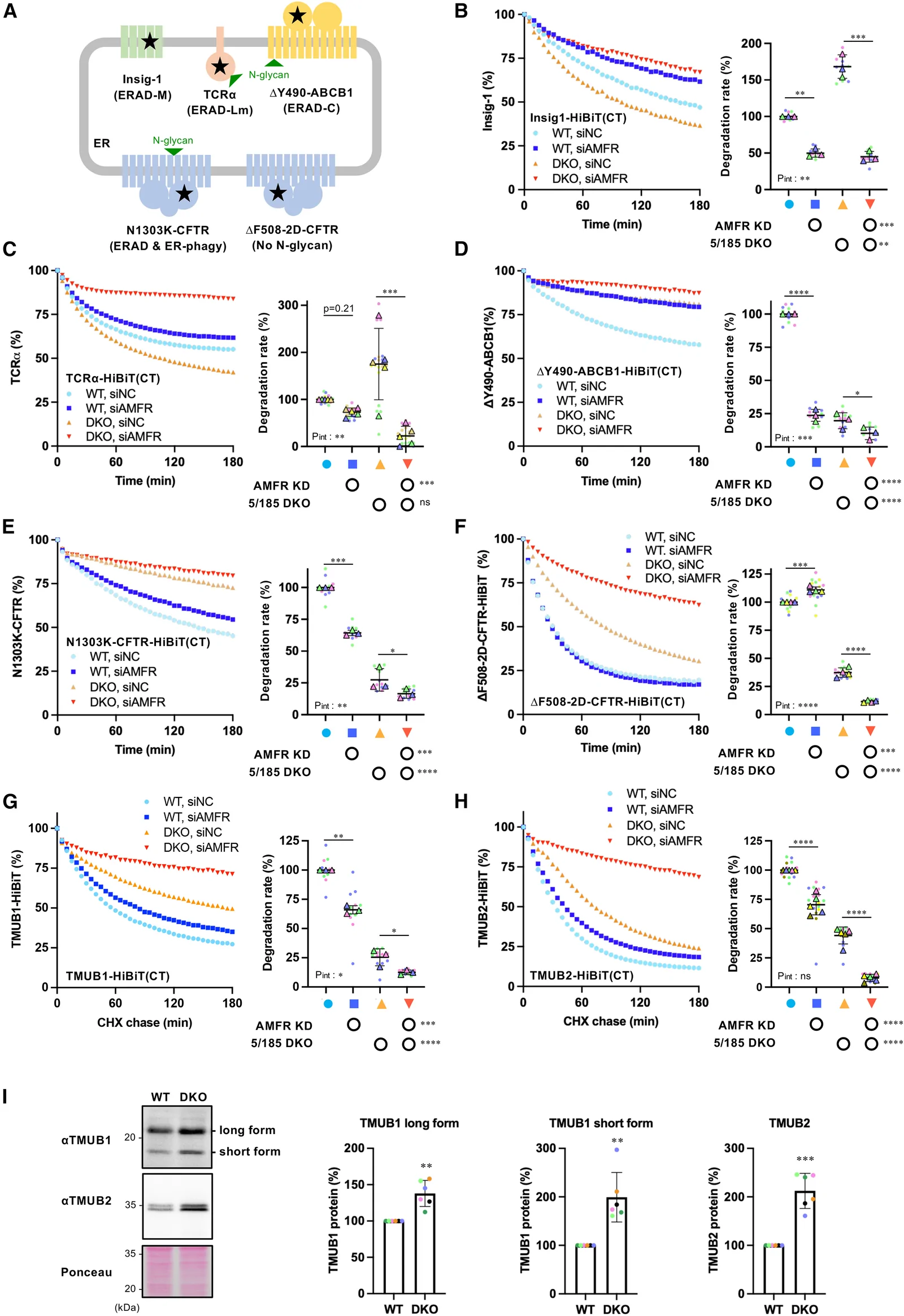
Effects of AMFR-mediated ERAD compensation on other ERAD substrates (A) Schematic representation of the ERAD substrate models. The misfolded regions are marked with stars, and the HiBiT tag was fused to the cytoplasmic domain. *N*-glycans are indicated for glycoproteins. (B–H) The HiBiT degradation assay was used to assess the ERAD of Insig1-HiBiT (B, *n* = 3), TCRα-HiBiT (C, *n* = 5), ΔY490-ABCB1-HiBiT (D, *n* = 3), N1303K-CFTR-HiBiT (E, *n* = 3), ΔF508-2D-CFTR-HiBiT (F, *n* = 4), TMUB1-HiBiT (G, *n* = 3), and TMUB2-HiBiT (H, *n* = 5) in 293MSR WT and RNF5/185 DKO cells transfected with 10 nM siNC or siAMFR-1, as described in Figure 2A. Each biological replicate (n) is color-coded, with the averages from four technical replicates represented by triangles. Statistical significance was determined using two-way RM ANOVA with Holm-Sidak multiple comparison tests. The analysis identified significant main effects of AMFR KD and/or RNF5/185 DKO (except for C) and significant statistical interactions between them in panels (B–G), but not in panel H (P_int_ < 0.05 for B-G; P_int_ > 0.05 for H). (I) Endogenous TMUB1 and TMUB2 protein levels in 293MSR WT and RNF5/185 DKO cells were measured by western blotting. Each biological replicate is color-coded. Statistical significance was determined using a two-tailed paired *t* test (*n* = 6). Data are presented as mean ± SD. ∗*p* < 0.05, ∗∗*p* < 0.01, ∗∗∗*p* < 0.001, ∗∗∗∗*p* < 0.0001, ns (not significant).

N1303K-CFTR, a misfolded CFTR mutant carrying an NBD2 mutation that destabilizes the membrane-spanning domains (MSD1 and MSD2),^54^ has been reported to be degraded through both ERAD and ER-phagy pathways.^55^ AMFR KD significantly reduced N1303K-CFTR degradation in WT cells, whereas this effect was less pronounced in RNF5/185 DKO cells (Figure 6E).

To determine whether the AMFR-mediated ERAD branch depends on *N*-glycan-dependent ERAD (GERAD),^56^^,^^57^ we analyzed ΔF508-2D-CFTR, in which the two *N*-glycosylation sites, N894 and N900, were mutated to aspartic acid residues (N894D and N900D) to abolish *N*-glycosylation.^58^ AMFR KD had comparable effects on the degradation of ΔF508-2D-CFTR and ΔF508-CFTR in both WT and RNF5/185 DKO cells (Figure 6F). These findings suggest that AMFR-mediated ΔF508-CFTR degradation is largely independent of *N*-glycosylation and is unlikely to depend primarily on the GERAD pathway.

Previous studies have shown that RNF185 forms a complex with TMUB1 and TMUB2,^59^ and that RNF5/185 DKO increases TMUB2 abundance.^60^ In addition, AMFR forms a complex with TMUB1, and the AMFR-TMUB1 complex is required for HMG-CoA reductase degradation.^61^ Based on these findings, we hypothesized that TMUB proteins function not only as accessory factors for RNF5/185 and AMFR, but also as ERAD-regulated substrates whose abundance is controlled by these ligases. Consistent with this hypothesis, HiBiT-based degradation assays revealed that both TMUB1 and TMUB2 were degraded through an ERAD-related pathway and that their degradation was reduced in RNF5/185 DKO cells (Figures 6G and 6H). AMFR KD also partially inhibited the degradation of TMUB1 and TMUB2 in WT cells and, importantly, this inhibitory effect was more pronounced in RNF5/185 DKO cells, particularly for TMUB2 (Figures 6G and 6H). These findings suggest that AMFR-mediated compensatory ERAD is not limited to ΔF508-CFTR but may also extend to other RNF5/185-regulated substrates, including TMUB2. Consistent with the degradation assay results, we also observed increased abundance of endogenous TMUB1 and TMUB2 proteins in RNF5/185 DKO cells compared with WT cells (Figure 6I). For TMUB1, both long and short isoforms, which differ in their translation start sites,^62^ were detected (Figure 6I).

To further investigate which regions of misfolded CFTR are preferentially associated with AMFR-dependent ERAD, we analyzed isolated CFTR domains. These isolated domains possess intrinsic conformational defects^54^ and are typically targeted for degradation by cellular protein QC mechanisms.^27^ The HiBiT assay demonstrated that AMFR KD had minimal impact on the degradation of CFTR MSD1 and MSD2 in WT cells. In contrast, in RNF5/185 DKO cells, AMFR KD significantly impaired the degradation of these domains (Figures 7A and 7B). However, AMFR KD did not significantly affect the degradation of ΔF508-CFTR NBD1 (Figure 7C). To further assess the physical interaction between AMFR and CFTR domains, we performed pull-down experiments in RNF5/185 DKO cells expressing HiBiT-tagged CFTR domains and AMFR fused to a Myc-biotin (MB) tag.^63^ Robust binding of AMFR was observed with CFTR MSD1 and MSD2, whereas its interaction with NBD1 was relatively weak (Figure 7D). Interestingly, high-molecular-weight smear bands were also detected in the AMFR precipitates (Figure 7D), suggesting that these bands may represent ubiquitinated CFTR forms. These findings suggest that AMFR preferentially interacts with CFTR transmembrane domains and contributes to their ERAD in the absence of RNF5/185.

**Figure 7:**
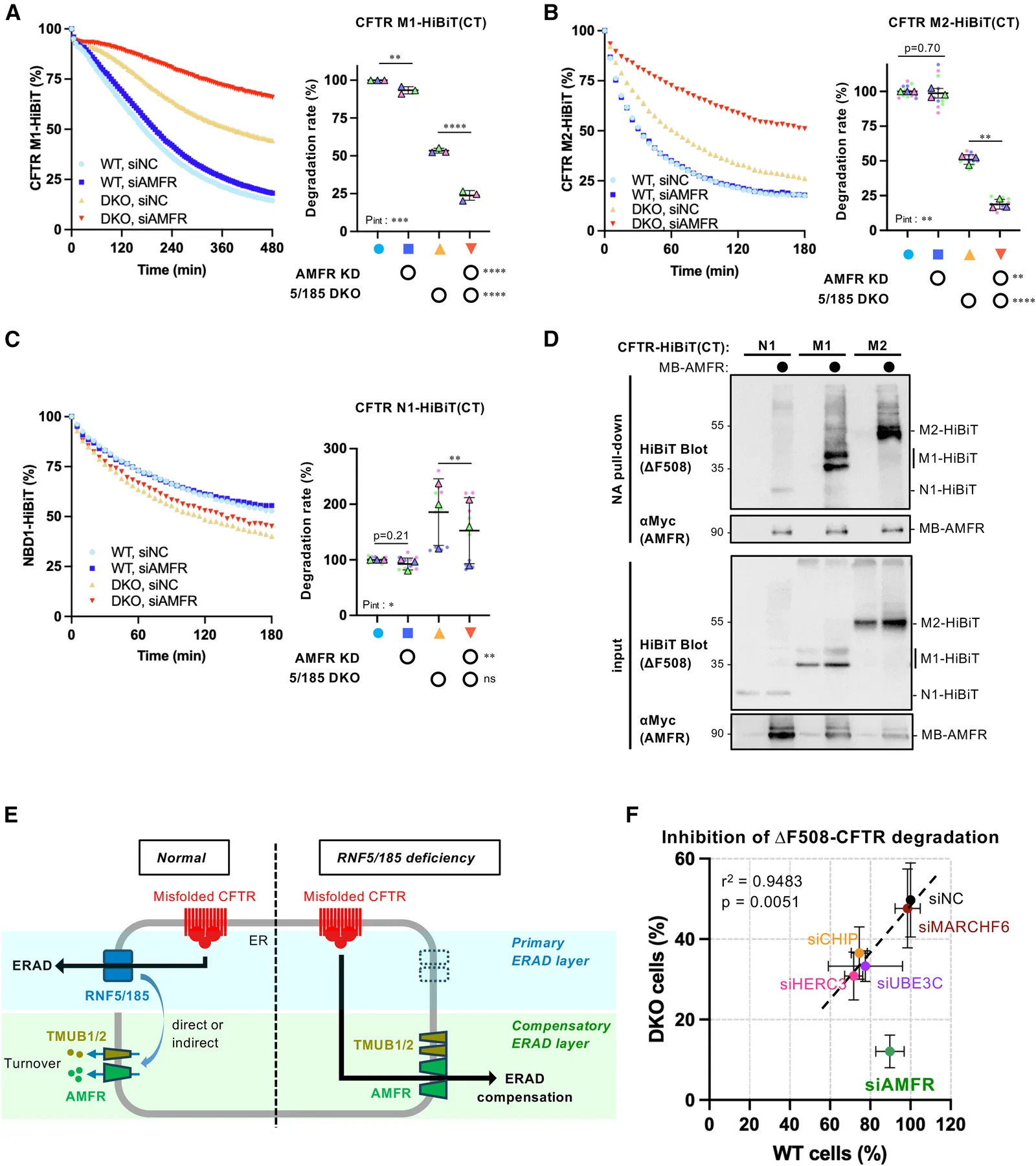
AMFR interacts with and facilitates ERAD of CFTR transmembrane domains (A–C) The HiBiT degradation assay was used to assess the ERAD of CFTR MSD1 (M1)-HiBiT (A, *n* = 3), MSD2 (M2)-HiBiT (B, *n* = 3), and NBD1 (N1)-HiBiT (C, *n* = 3) in 293MSR WT and RNF5/185 DKO cells transfected with 10 nM siNC or siAMFR-1, as described in Figure 2A. Each biological replicate (n) is color-coded, with the averages from four technical replicates represented by triangles. Statistical significance was determined using two-way RM ANOVA with Holm-Sidak multiple comparison tests. Data are presented as mean ± SD. ∗*p* < 0.05, ∗∗*p* < 0.01, ∗∗∗*p* < 0.001, ∗∗∗∗*p* < 0.0001, ns (not significant). (D) The interaction between MB-AMFR and co-expressed CFTR N1-, M1-, and M2-HiBiT in RNF5/185 DKO cells was analyzed by NA pull-down followed by western blotting. Cells were pre-treated with 10 μM MG-132 for 3 h at 37°C before lysis. The HiBiT-fused CFTR domain proteins were detected using the HiBiT Blotting system, while MB-AMFR was detected with an anti-Myc antibody. (E) Proposed model of the AMFR-mediated compensatory ERAD system. Under normal conditions (e.g., in WT cells), the RNF5/185-dependent ERAD branch primarily eliminates misfolded CFTR (ΔF508-CFTR). When RNF5/185 function is impaired (e.g., in RNF5/185 DKO cells), AMFR turnover is reduced, which is associated with increased AMFR protein abundance. TMUB1/2 protein levels are also increased under these conditions. These changes coincide with a greater contribution of AMFR-dependent ERAD to misfolded CFTR degradation. (F) Correlation analysis of the effects of ERAD-associated E3 ligase KD on ΔF508-CFTR-HiBiT degradation rates in 293MSR WT and RNF5/185 DKO cells. Quantitative data from Figures 2A, 5A–5C, and S3L were used for this analysis. AMFR is highlighted as an outlier. Outlier detection was performed using the ROUT method (Q = 1%) in GraphPad Prism. A single data point (AMFR) was identified as an outlier and excluded before linear regression analysis. Linear regression was then performed on the remaining data points, and the coefficient of determination (R^2^) and *p* value were calculated to assess the strength and significance of the correlation.

## Discussion

This study demonstrates that, among the ER-associated E3 ligases examined, AMFR plays a distinct role in compensatory CFTR ERAD when the major RNF5/185-dependent ERAD pathway is impaired. We further show that AMFR protein abundance is increased and its turnover is reduced in RNF5/185-deficient cells. Based on these findings, we propose an adaptable model of ER proteostasis in which different E3 ligase branches can make greater functional contributions when a major pathway is impaired (Figure 7E). The compensatory AMFR pathway is unlikely to represent a pathway distinct from canonical ERAD; instead, it reflects increased reliance on an AMFR-dependent ERAD branch when RNF5/185-dependent ERAD activity is reduced. One possibility is that RNF5/185 normally promotes AMFR turnover, either directly or indirectly through other ERAD/proteostasis regulatory factors, such that loss of RNF5/185 stabilizes AMFR. Alternatively, or additionally, chronic RNF5/185 deficiency may indirectly remodel AMFR expression and other proteostasis pathways. Consistent with the latter possibility, AMFR mRNA showed an upward trend in RNF5/185 DKO cells, although the difference did not reach statistical significance. Thus, the increased AMFR abundance may reflect a combination of enhanced protein stability and transcriptional or post-transcriptional regulation. Increased AMFR abundance may, in turn, contribute to the enhanced functional importance of AMFR-dependent ERAD in RNF5/185 DKO cells (Figure 7E). Together with previous findings that AMFR overexpression promotes CFTR ERAD,^29^ these observations provide a plausible basis for increased AMFR-dependent ERAD capacity, although the causal relationship between RNF5/185 deficiency and increased AMFR abundance remains to be established.

AMFR has previously been proposed to facilitate ERAD of misfolded CFTR as an E4-like enzyme downstream of RNF5.^29^ In our study, AMFR KD had a smaller effect on the C-terminal HiBiT degradation readout in WT cells than on several cytosolic-accessibility, ubiquitination, and steady-state abundance readouts. These assays interrogate different molecular steps and CFTR pools and should therefore be regarded as complementary rather than interchangeable measures of CFTR quality control. In particular, the C-terminal HiBiT assay primarily monitors the disappearance of a fully synthesized CFTR pool after CHX treatment, whereas the other assays probe earlier or distinct ERAD-associated steps. In WT cells, a reduction in an early extraction- or ubiquitination-associated readout caused by AMFR KD may be buffered by the remaining RNF5/185-dependent ERAD capacity and therefore may not produce a proportionally large change in overall CFTR degradation. This provides a plausible explanation for the relatively small effect of AMFR KD on the C-terminal HiBiT degradation readout despite clearer effects on other ERAD-associated measurements.

Although AMFR KD strongly reduced CFTR pan-ubiquitination in RNF5/185 DKO cells, its effect on K48-linked ubiquitination was relatively modest. This suggests that AMFR-dependent CFTR ERAD in RNF5/185-deficient cells may involve Ub-chain architectures beyond canonical K48-linked chains. ERAD substrates can be modified by non-K48 linkages, mixed chains, and branched chains, which may influence substrate extraction, recognition by Ub-binding adaptors, and proteasomal degradation.^38^ In line with this possibility, our Ub-linkage analysis showed that AMFR KD tended to reduce multiple Ub linkages on CFTR in RNF5/185 DKO cells, including K29- and K63-linked chains as well as K48-linked chains. These findings suggest that AMFR may support compensatory CFTR ERAD by modulating the broader ubiquitination landscape of CFTR, rather than exclusively promoting K48-linked ubiquitination. This interpretation reconciles the strong effect of AMFR KD on CFTR degradation in RNF5/185 DKO cells with its relatively modest effect on K48-linked ubiquitination. Although AMFR has also been implicated in ER-phagy,^34^^,^^35^ our pharmacological data indicate that ΔF508-CFTR degradation in RNF5/185 DKO cells is predominantly proteasome dependent. Nevertheless, the partial effect of lysosomal inhibition suggests that lysosome-dependent pathways, potentially including ER-phagy, may also contribute to ΔF508-CFTR turnover under these conditions. Thus, the AMFR-dependent compensatory pathway described here is best interpreted as a proteasome-dependent ERAD pathway, while a contribution from ER-phagy cannot be fully excluded.

Notably, a comparable AMFR-like compensatory effect on ERAD was not observed with other ER-associated E3 ligases, including SYVN1, RNF139, and RNF145, which are involved in multiple ERAD pathways.^1^^,^^2^^,^^3^^,^^4^^,^^6^^,^^8^^,^^15^^,^^43^^,^^44^^,^^45^^,^^46^^,^^47^^,^^48^^,^^49^^,^^50^ Consistent with our model, correlation analysis of the inhibitory effects on ΔF508-CFTR ERAD in WT versus RNF5/185 DKO cells revealed strong concordance for KD of CHIP, HERC3, UBE3C, and MARCHF6, whereas AMFR KD displayed a distinct profile, showing modest effects in WT cells but markedly inhibiting ERAD under RNF5/185-deficient conditions, indicating a distinct compensatory function (Figure 7F). Thus, among the E3 ligases examined, AMFR showed a distinct shift in its functional contribution upon RNF5/185 deficiency. Distinct functional roles of AMFR and SYVN1 have been reported previously, and our results are consistent with these observations.^41^^,^^64^^,^^65^ Although SYVN1 KD modestly increased steady-state ΔF508-CFTR levels and reduced CFTR ubiquitination in WT cells, it did not significantly suppress post-translational CFTR ERAD in the HiBiT-based degradation assay or increase PM CFTR expression. Thus, SYVN1 may partially contribute to early quality-control steps of ΔF508-CFTR, but it does not appear to function as a compensatory ERAD ligase when the RNF5/185-dependent pathway is impaired.

While AMFR is primarily associated with the ERAD-M pathway, our findings suggest that it can compensate for dysfunction of the ERAD-C pathway, which is typically mediated by RNF5/185. Although ΔF508-CFTR is generally classified as an ERAD-C substrate,^23^^,^^24^ structural abnormalities within its transmembrane domains cannot be excluded, given that ΔF508-CFTR correctors are known to bind these regions.^66^ Indeed, structural defects in the CFTR MSDs have been identified by *in situ* trypsin sensitivity assays.^54^ Furthermore, our observation that AMFR regulates the degradation of isolated CFTR MSD1/2 supports the notion that ΔF508-CFTR harbors defects in both cytosolic and membrane-embedded regions. Consistent with this, AlphaFold3-based structural predictions suggest that RNF5/185 may directly engage the transmembrane region of CFTR, particularly TM1 (Figure S4A), indicating that RNF5/185 may function beyond canonical ERAD-C and contribute to ERAD-M-like substrate recognition. This is further supported by previous findings that RNF185 can recognize the transmembrane domain of CYP51A1 and facilitate its ERAD.^59^ Together, these observations raise the possibility that RNF5/185 regulates CFTR degradation through both ERAD-C and ERAD-M mechanisms, resembling the dual role of the yeast E3 ligase Doa10, which participates in both pathways.^4^^,^^6^^,^^11^^,^^15^ In parallel, AMFR is also predicted to interact with CFTR TM1 (Figure S4B), providing a possible structural basis for functional compensation between these ligases. Accordingly, AMFR may recognize transmembrane defects in CFTR and compensate for RNF5/185 function when its activity is compromised or limiting.

Although the physiological relevance of AMFR-mediated ERAD compensation remains to be fully defined, this pathway may become particularly important under pathological conditions associated with reduced RNF5 activity, such as myocardial infarction or ischemia.^67^ Such compensation may function as a fail-safe mechanism that promotes the clearance of highly proteotoxic species. Given that AMFR has been implicated in the attenuation of ER stress^68^ and in the degradation of diverse substrates,^69^^,^^70^^,^^71^ AMFR-dependent compensatory ERAD may help preserve ER proteostasis when the RNF5/185 pathway is impaired. Recent studies showing that AMFR contributes to ΔF508-CFTR degradation in RNF5-deficient cells^30^ and that AMFR KD inhibits TAP2 degradation in RNF5/185 DKO cells^62^ support the broader relevance of this mechanism. In addition, our finding that AMFR KD markedly inhibited TMUB2 degradation in RNF5/185-deficient cells suggests that its substrate range may extend beyond CFTR. TMUB1 and TMUB2 may also contribute to this compensatory context because they are AMFR-associated auxiliary factors that facilitate degradation of AMFR substrates,^61^ and their endogenous levels were increased in RNF5/185 DKO cells. Increased TMUB1/2 abundance has also been reported previously in RNF185-deficient cells.^59^^,^^60^ These observations raise the possibility that changes in both AMFR and its associated factors contribute to compensatory ERAD capacity.

CFTR degradation proceeded more rapidly in BEAS-2B airway epithelial cells than in non-epithelial 293MSR cells, consistent with our previous findings in epithelial cell models, including BEAS-2B and CFBE cells.^27^ The effects of AMFR or RNF5/185 KD were weaker in BEAS-2B cells than in 293MSR cells, likely due in part to lower KD efficiency in BEAS-2B cells. Airway epithelial cells may also possess more stringent ER quality-control mechanisms and/or additional compensatory degradation pathways, including the HERC3 E3 ligase pathway.^27^ These cell-type differences suggest that the contribution of individual ERAD ligases may vary depending on cellular context, proteostasis demand, and the available quality-control network.

Unlike in yeast, ERAD pathways in mammalian cells are highly diversified and difficult to dissect because of overlapping and context-dependent functions.^2^^,^^8^^,^^71^ By combining E3 ligase KO/KD approaches with quantitative degradation measurements, our study provides a framework for distinguishing independent, overlapping, and compensatory ERAD pathways in mammalian cells. Overall, our findings support a model in which mammalian ERAD is adaptable, with the functional contribution of individual E3 ligases depending on the state of the surrounding proteostasis network.

### Limitations of the study

Several limitations remain. Most experiments were performed in cultured cell models, and future studies in physiologically relevant tissues or *in vivo* models will be needed to define when and where AMFR-mediated compensatory ERAD operates. In addition, although our data suggest that this pathway extends beyond CFTR to substrates such as TMUB2, its full substrate range and specificity remain unclear. The AlphaFold3-based structural models also require experimental validation by biochemical, mutational, or structural approaches. Finally, chronic RNF5/185 deficiency may alter additional E3 ligases or ERAD-related factors that contribute to residual degradation together with AMFR or that indirectly regulate AMFR abundance. The causal mechanism linking RNF5/185 deficiency to increased AMFR abundance remains unresolved. Future unbiased proteomic analyses will therefore be important for defining the broader remodeling of the ERAD network caused by reduced RNF5/185 activity.

## Resource availability

### Lead contact

Further information and requests for resources and reagents may be directed to and will be fulfilled by Tsukasa Okiyoneda (t-okiyoneda@kwansei.ac.jp).

### Materials availability

Plasmids and cell lines generated in this study are available from the lead contact upon reasonable request.

### Data and code availability

• All data reported in this paper will be shared by the lead contact upon request.
• The raw data required to reproduce these findings are available from the corresponding authors upon reasonable request. The RNA-seq datasets generated in this study have been deposited in the NCBI Gene Expression Omnibus (GEO) under accession number GSE334332 (https://www.ncbi.nlm.nih.gov/geo/query/acc.cgi?acc=GSE334332) and are publicly available without restrictions. Data S1 contains uncropped western blot images. This paper does not report original code. Any additional information required to reanalyze the data reported in this paper is available from the lead contact upon request.

## Acknowledgments

We thank R. Kopito (Stanford University) and Y. Ye (NIH/NIDDK) for providing TCRα-HA, N. Hosokawa (Kyoto University) for mouse AMFR, H. Takahashi (Ehime University) for TMUB1 and TMUB2, and D. Root for providing expression vectors (Addgene #41394, pLIX402; #25890, pLX304). We also acknowledge support for RNA-seq analysis from Y. Mizukami and K. Watanabe (Yamaguchi University), and thank M. Okumura and S. Kanemura (Tohoku University) for valuable discussions on structural analyses. This work was supported by JSPS/MEXT KAKENHI (22H02576, 25K02231 to T.O.; 24K23193, 25K18425 to Y. Kamada) and the Individual Special Research Subsidy with grants from Kwansei Gakuin University (to T.O.).

## Author contributions

Conceptualization, T.O.; methodology, U.N., A.F., Y.Kamada; investigation, U.N., A.F., S.K., Y.Karasaki, M.N., N.M., M.Y., Y.Kamada; data curation, U.N., A.F., S.K.; formal analysis, T.O.; writing – original draft, U.N., A.F., T.O.; writing – review and editing, Y.Kamada, T.O.; visualization, T.O.; funding acquisition, Y.Kamada, T.O.; resources, T.O.; supervision, T.O.

## Declaration of interests

The authors declare no competing interests.

## Declaration of generative AI and AI-assisted technologies in the manuscript preparation process

During the preparation of this work the authors used ChatGPT to improve language and readability. After using this tool/service, the authors reviewed and edited the content as needed and take full responsibility for the content of the publication.

## STAR★Methods

### Key resources table

**Table:** 

REAGENT or RESOURCE	SOURCE	IDENTIFIER
**Antibodies**		

HA (16B12)	BioLegend	Cat#901515; RRID:AB_2565334
AMFR	Cell Signaling Technology	Cat#9590; RRID:AB_10860080
SYVN1	Cell Signaling Technology	Cat#14773; RRID:AB_2798607
RNF185 antiserum	Kamada et al.^27^	N/A
CHIP (H-231)	Santa Cruz Biotechnology	Cat#sc-66830; RRID:AB_2198073
UBE3C	abcepta	Cat#AP20457c
Ub (P4D1)	Santa Cruz Biotechnology	Cat#sc-8017; RRID:AB_628423
Myc (9E10)	FUJIFILM Wako	Cat#017–21871
K48-Ub (Apu2)	Merck Millipore	Cat#05–1307; RRID:AB_1587578
TMUB1	Proteintech	Cat#16638-1-AP; RRID:AB_3085503
TMUB2	Proteintech	Cat#28044-1-AP; RRID:AB_2881045
Peroxidase AffiniPure Goat Anti-Mouse IgG (H + L)	Jackson Immuno Research	Cat#115–035–166; RRID:AB_2338511
Peroxidase AffiniPure Donkey Anti- Rabbit IgG (H + L)	Jackson Immuno Research	Cat#711–035–152; RRID:AB_10015282

**Chemicals, peptides, and recombinant proteins**		

Lipofectamine™ RNAiMAX Transfection Reagent	Invitrogen	Cat#13778150
Doxycycline Hydrochloride n-Hydrate (Dox)	FUJIFILM Wako	Cat#049–31121
Cycloheximide (CHX)	FUJIFILM Wako	Cat#037–20991
MG-132	Cayman	Cat#10012628
Bortezomib	FUJIFILM Wako	Cat#021–18901
Bafilomycin A1	Cayman	Cat#11038
N-Ethylmaleimide (NEM)	FUJIFILM Wako	Cat#054–02063
Pepstatin A	Peptide institute	Cat#4397
Leupeptin Hemisulfate monohydrate	FUJIFILM Wako	Cat#122–03751
Benzylsulfonyl Fluoride (PMSF)	FUJIFILM Wako	Cat#164–12181
Ponceau-S	Sigma-Aldrich	Cat#24–3875–5
Ponceau-S	Beacle, Inc	Cat#BCL-PSS-01
NeutrAvidin agarose	ThermoFisher	Cat#29200
Nunc™ FluoroNunc™/LumiNunc™ 96-Well Plates	ThermoFisher	Cat#437796
Avidin, NeutrAvidin™ Biotin-binding Protein	ThermoFisher	Cat#A2666
Nunc™ MicroWell™ 96-Well, Nunclon Delta-Treated, Flat-Bottom Microplate	ThermoFisher	Cat#136101
Albumin, from Bovine Serum, Fraction V pH7.0 (BSA)	FUJIFILM Wako	Cat#015–27053
(+)-Biotin	FUJIFILM Wako	Cat#029–08713
Urea	FUJIFILM Wako	Cat#217–00615
CO_2_ independent medium	ThermoFisher	Cat#18045088
Polyethylenimine (PEI) Max	Polyscience, Inc	Cat#24765
G418	FUJIFILM Wako	Cat#074–05963
puromycin	Sigma-Aldrich	Cat#P9620-10 ML
Blasticidin S, Hydrochloride	Funakoshi	Cat#KK-400

**Critical commercial assays**		

Nano-Glo® Endurazine™ Live Cell Substrate	Promega	Cat#N2571
Extracellular NanoLuc® Substrate	Promega	Cat#CS3135
Nano-Glo® HiBiT Blotting System	Promega	Cat#N2410
SuperSignal West Pico PLUS Chemiluminescent Substrate	ThermoFisher	Cat#34580
ImmunoStar® Zeta	FUJIFILM Wako	Cat#295–72404
ImmunoStar LD	FUJIFILM Wako	Cat#292–69903

**Experimental models: Cell lines**		

293MSR (WT)	ThermoFisher	Cat#R79507; RRID:CVCL_U428
293MSR RNF5/185 DKO (RNF5/185 DKO)	Kamada et al.^27^	N/A
293MSR HBH-ΔF508-CFTR-3HA	Kamada et al.^27^	N/A
293MSR RNF5/185 DKO HBH-ΔF508-CFTR-3HA	Kamada et al.^27^	N/A
293MSR teton ΔF508-CFTR-Nluc(Ex)	This paper	N/A
293MSR RNF5/185 DKO teton ΔF508-CFTR-Nluc(Ex)	This paper	N/A
293MSR teton ΔF508-CFTR-3HA-Nluc(CT)	This paper	N/A
293MSR RNF5/185 DKO teton ΔF508-CFTR-3HA-Nluc(CT)	This paper	N/A
BEAS-2B	ECACC	Cat#95102433; RRID:CVCL_0168
BEAS-2B teton ΔF508-CFTR-3HA-Nluc(CT)	Kamada et al.^27^	N/A
BEAS-2B ΔF508-CFTR-HiBiT(Ex)	Taniguchi et al.^32^	N/A

**Oligonucleotides**		

hs.Ri.AMFR.13.1 (siAMFR-1)	IDT	Cat#319764314
hs.Ri.AMFR.13.2 (siAMFR-2)	IDT	Cat#319764317
hs.Ri.AMFR.13.3 (siAMFR-3)	IDT	Cat#319764320
hs.Ri.AMFR.13.4 (siAMFR-4)	IDT	Cat# 466396869
hs.Ri.UBR4.13.1 (siUBR4-1)	IDT	Cat#329542790
hs.Ri.UBR4.13.3 (siUBR4-3)	IDT	Cat#329542793
hs.Ri.CUL7.13.1 (siCUL7-1)	IDT	Cat#329542796
hs.Ri.CUL7.13.3 (siCUL7-3)	IDT	Cat#329542799
hs.Ri.MARCH6.13.2 (siMARCHF6-2)	IDT	Cat#329542745
hs.Ri.MARCH6.13.3 (siMARCHF6-3)	IDT	Cat#508008684
hs.Ri.RNF122.13.1 (siRNF122-1)	IDT	Cat#329542748
hs.Ri.RNF122.13.2 (siRNF122-2)	IDT	Cat#329542751
hs.Ri.RNF139.13.1 (siRNF139-1)	IDT	Cat#329542730
hs.Ri.RNF139.13.3 (siRNF139-3)	IDT	Cat#329542733
hs.Ri.RNF145.13.1 (siRNF145-1)	IDT	Cat#329542736
hs.Ri.RNF145.13.2 (siRNF145-2)	IDT	Cat#329542739
hs.Ri.RNF157.13.1 (siRNF157-1)	IDT	Cat#329542808
hs.Ri.RNF157.13.3 (siRNF157-3)	IDT	Cat#329542811
hs.Ri.RNF170.13.1 (siRNF170-1)	IDT	Cat#329542724
hs.Ri.RNF170.13.2 (siRNF170-2)	IDT	Cat#329542727
hs.Ri.RNFT1.13.1 (siRNFT1-1)	IDT	Cat#329542766
hs.Ri.RNFT1.13.2 (siRNFT1-2)	IDT	Cat#329542769
hs.Ri.TMEM129.13.1 (siTMEM129-1)	IDT	Cat#329542772
hs.Ri.TMEM129.13.3 (siTMEM129-3)	IDT	Cat#329542775
hs.Ri.TMEM259.13.1 (siTMEM259-1)	IDT	Cat#329542778
hs.Ri.TMEM259.13.2 (siTMEM259-2)	IDT	Cat#329542781
hs.Ri.UBR5.13.1 (siUBR5-1)	IDT	Cat#329542784
hs.Ri.UBR5.13.3 (siUBR5-3)	IDT	Cat#329542787
hs.Ri.SYVN1.13.1 (siSYVN1-1)	IDT	Cat#308952672
hs.Ri.SYVN1.13.4 (siSYVN1-4)	IDT	Cat#466396875
hs.Ri.UBE3C.13.1 (siUBE3C)	IDT	Cat#439958530
hs.Ri.RNF213.13.2 (siRNF213-2)	IDT	Cat#579003262
hs.Ri.RNF150.13.1 (siRNF150-1)	IDT	Cat# 579003265
Negative Control DsiRNA (siNC)	IDT	Cat#110644596
AllStars Neg. Control siRNA (siNT)	Qiagen	Cat#1027281
Hs_RNF5_11 siRNA: CAGCCAGAGAAGAATCAGTAT	Qiagen	Cat#SI05063114
Hs_RNF5_6 siRNA: GGGCGCGACCTTCGAATGTAA	Qiagen	Cat#SI03221484
Hs_RNF5_7 siRNA: AACGGCAAGAGTGTCCAGTAT	Qiagen	Cat#SI04204116
Hs_RNF185_2 siRNA: CACCTTCTGGAAATCCGCAAA	Qiagen	Cat#SI03059385
Hs_RNF185_3 siRNA: CTCCGAGGCAGTAATAACTGA	Qiagen	Cat#SI03089982
Hs_RNF185_8 siRNA: TCCCATCAGCTTTCTCCCTAA	Qiagen	Cat#SI04959598
Hs_STUB1 (CHIP)_1 siRNA: CCCGCTGGTGGCCGTGTATTA	Qiagen	Cat#SI00081963
Hs_STUB1 (CHIP)_2 siRNA: CCAGCTGGAGATGGAGAGCTA	Qiagen	Cat#SI00081970
Hs_STUB1 (CHIP)_5 siRNA: TACGCGAACGCCCACCCTTAA	Qiagen	Cat#SI03109659
Hs_Hs_HERC3_4 siRNA: CAGCCCGAGCTGATCGCTTTA	Qiagen	Cat#SI00107233
Hs_Hs_HERC3_5 siRNA: CAGCGATCGGATTCCCATCTA	Qiagen	Cat#SI03066959
Hs_Hs_HERC3_8 siRNA: TTGAGTCTCTATATTGTCCAA	Qiagen	Cat#SI05092101
AMFR qPCR primer pair: 5′-TTCCACAACTCCTGTCTTCG-3' (FW) 5′-CTGCTGCTACAGGAACCAAA-3' (Rev)	This paper	N/A
HERC3 qPCR primer pair: 5′-GATCTGCCGAGAAAGCTATGG-3' (FW) 5′-TTTGCCACCACACACCTTTAG-3' (Rev)	Kamada et al.^27^	N/A
RNF170 qPCR primer pair: 5′-TGGCAGTTGTGGTCAGTTTCGC-3' (FW) 5′-CGAAGTACCCTTACTAGCTCCTG-3' (RV)	This paper	N/A
RNF139 qPCR primer pair: 5′-TGCATCGTGCTCCAGATCTTCC-3' (FW) 5′-CAGAAAGGCTGAGCTGTACGTG-3' (RV)	This paper	N/A
RNF145 qPCR primer pair: 5′-GCAGGTTGTTCATCGGGCATTC-3' (FW) 5′-GGCTTACAGCACGGAAGTGTTTC-3' (RV)	This paper	N/A
TMEM129 qPCR primer pair: 5′-GAGACATTTGCCTCCCTGGTAG-3' (FW) 5′-TGGCAGGTCTTCACCAGCTTCA-3' (RV)	This paper	N/A
RNF122 qPCR primer pair: 5′-TCCAGGACCTTCCGCTCAACAT-3' (FW) 5′-ATCCGTATCGCTCACTCTGTGC-3' (RV)	This paper	N/A
CUL7 qPCR primer pair: 5′-CCGCAAACTCATCACCAACATCC-3' (FW) 5′-GGCACAGGTATCTGAGGAACAC-3' (RV)	This paper	N/A
RNFT1 qPCR primer pair: 5′-TGGAGTCTTGGGATACTGCTGG-3' (FW) 5′-TCTTGCTGGCAGCCACTCCATA-3' (RV)	This paper	N/A
RNF157 qPCR primer pair: 5′-CTCACCTTGTCGTCATCTGGAG-3' (FW) 5′-AGACGGTGTCAGTGCTGATCTG-3' (RV)	This paper	N/A
RNF213 qPCR primer pair: 5′-GGAAAGGAAACCTCTGAACTCGG-3' (FW) 5′-CTCGTTCTGGTCTCTGAGCATG-3' (RV)	This paper	N/A
RNF150 qPCR primer pair: 5′-TCAGCCGTGGTCATCTTCAACG-3' (FW) 5′-CGGTGATGTTTCTTTCCAGCAGG-3' (RV)	This paper	N/A
MARCHF6 qPCR primer pair: 5′-GCTCACTACTGACGCTGCCATT-3' (FW) 5′-CTCTCAACCACACCAGGCTGAT-3' (RV)	This paper	N/A
GAPDH qPCR primer pair: 5′-CATGAGAAGTATGACAACAGCCT-3' (FW) 5′-AGTCCTTCCACGATACCAAAGT-3' (Rev)	Kamada et al.^27^	N/A

**Recombinant DNA**		

pNUT-HBH-ΔF508-CFTR-3HA	Okiyoneda et al.^36^	N/A
pNUT ΔF508-CFTR-3HA-HiBiT(CT)	Kamada et al.^27^	N/A
pBiT1.1-N [TK/LgBiT]	Promega	Cat#N198A
pBiT1.1-N [TK/CNXss-LgBiT-KDEL]	Kamada et al.^27^	N/A
pcDNA6 ΔF508-CFTR-HiBiT(Ex)	Kamada et al.^27^	N/A
pLX303 HBH-ΔF508 CFTR-3HA	Kamada et al.^27^	N/A
pLIX402	Addgene	Cat#41394
pLIX402 blast	This paper	N/A
pLIX402 ΔF508-CFTR-Nluc(Ex)	Taniguchi et al.^31^	N/A
pLIX402 Nluc(CT)	Kamada et al.^27^	N/A
pLIX402 ΔF508-CFTR-3HA-Nluc(CT)	Kamada et al.^27^	N/A
pBiT2.1 TCRα-HiBiT(CT)	Kamada et al.^27^	N/A
pBiT2.1 Insig-1-HiBiT	Kamada et al.^27^	N/A
pBiT2.1 ΔY490-ABCB1-HiBiT	Kamada et al.^27^	N/A
pNUT CFTR M1 (1–413 aa)-HiBiT	Kamada et al.^27^	N/A
pNUT CFTR M2 (733–1218 aa)-3HA-HiBiT	Kamada et al.^27^	N/A
pcDNA3.1 mouse AMFR (gp78)-/Myc	Morito et al.^29^	N/A
pcDNA-Myc-Bio(MB)-mouse AMFR (gp78)	This paper	N/A
pNUT N1303K-CFTR-3HA-HiBiT(CT)	Kamada et al.^27^	N/A
pNUT ΔF508-2D-CFTR-3HA-HiBiT(CT)	This paper	N/A
pLIX402 blast TMUB1 HiBiT(CT)	This paper	N/A
pLIX402 blast TMUB2 HiBiT(CT)	This paper	N/A
pcDNA3.1(−)	ThermoFisher	Cat#V79520
pcDNA3.1(−) Myc-Ubiquitin	This paper	N/A
pcDNA3.1(−) Myc-K11 Ubiquitin	This paper	N/A
pcDNA3.1(−) Myc-K29 Ubiquitin	This paper	N/A
pcDNA3.1(−) Myc-K33 Ubiquitin	This paper	N/A
pcDNA3.1(−) Myc-K48 Ubiquitin	This paper	N/A
pcDNA3.1(−) Myc-K63 Ubiquitin	This paper	N/A

**Deposited data**		

RNA-seq datasets	This paper	GSE334332

**Software and algorithms**		

GraphPad Prism 8	GraphPad Software, Inc	RRID:SCR_002798
CLC Genomics Workbench	Qiagen	RRID:SCR_011853
Evolution Capt	Vilber-Lourmat	N/A
ImageQuant	Cytiva	RRID:SCR_014246
AlphaFold 3	Google DeepMind and Isomorphic Labs	RRID:SCR_025885
PyMOL	Schrödinger, LLC	RRID:SCR_000305

### Experimental model and study participant details

#### Cell lines

293MSR cells (GripTite 293 MSR, female; RRID: CVCL_U428) were obtained from Thermo Fisher Scientific. These cells, RNF5/185 DKO 293MSR cells, and their respective transfectants were cultured as previously described.^27^ Tet-on derivatives of 293MSR WT and RNF5/185 DKO cells stably expressing ΔF508-CFTR-Nluc(Ex) or ΔF508-CFTR-3HA-Nluc(CT) were generated via lentiviral transduction, as previously reported,^32^^,^^36^ and maintained in DMEM supplemented with 10% fetal bovine serum (FBS), 0.5 mg/mL G418, and 5 μg/mL blasticidin S. The human bronchial epithelial cell line BEAS-2B (male; RRID:CVCL_0168) was obtained from the European Collection of Authenticated Cell Cultures (ECACC; Culture Collections, UK Health Security Agency, Salisbury, UK). BEAS-2B Tet-on ΔF508-CFTR-3HA-Nluc(CT) cells were cultured following previously established protocols.^27^ CFTR expression in 293MSR and BEAS-2B cells was induced by treatment with 1 μg/mL doxycycline (Dox) for 2 days (293MSR) or 4 days (BEAS-2B), unless otherwise specified. All cell culture media were supplemented with 100 U/ml penicillin and 100 μg/mL streptomycin (FUJIFILM Wako Pure Chemical Corporation), and cells were maintained at 37°C in 5% CO_2_. No independent cell-line authentication was performed in this study. The influence of biological sex on the experimental outcomes was not assessed because the study was not designed to compare male- and female-derived cell lines. All cell lines tested negative for mycoplasma contamination.

### Method details

#### Materials and methods

##### Plasmids and antibodies

Plasmids, chemicals, and antibodies used in this study are listed in the key resources table. pcDNA3.1(−)-Myc–ubiquitin (WT) and its single-lysine (K) mutants, in which only one lysine residue is retained at the specified position, were constructed using PCR-based cloning methods. pNUT ΔF508-2D-CFTR-3HA-HiBiT(CT) was generated by introducing N-glycosylation site mutations (N894D and N900D) into pNUT ΔF508-CFTR-3HA-HiBiT(CT),^27^ as previously described.^58^ TCRα-HiBiT was constructed by inserting TCRα from pCMV-TCRα-HA (a kind gift from R. Kopito) into the pBiT2.1-HiBiT vector (Promega) via In-Fusion cloning. MB-AMFR was generated by subcloning mouse AMFR from pcDNA3.1 (a kind gift from N. Hosokawa) into the pcDNA-Myc-Bio Gateway destination vector^63^ using Gateway cloning. pLIX402-TMUB1-HiBiT and pLIX402-TMUB2-HiBiT were constructed by inserting TMUB1 and TMUB2 cDNAs (a kind gift from H. Takahashi) into the pLIX402-HiBiT vector via Gateway cloning.

##### Mammalian cell transfection

Transient transfection in 293MSR and RNF5/185 DKO cells was performed using polyethylenimine (PEI) Max (Polysciences Inc.), and experiments were conducted 2 days post-transfection. siRNA transfections (typically at 10 nM) were carried out using Lipofectamine RNAiMax (Thermo Fisher Scientific) following the manufacturer’s instructions. Unless otherwise specified, siRNA-transfected cells were used for experiments 4 days post-transfection. The siRNAs used in this study are listed in the key resources table. Unless explicitly stated, pooled siRNA was used for KD. The pooled siRNA was prepared by mixing equal amounts of two or three individual siRNAs, as listed in the key resources table. Negative control siRNAs (siNC) were purchased from Integrated DNA Technologies (IDT, Coralville, IA, USA) and Qiagen (Venlo, the Netherlands; AllStars Negative Control siRNA).

##### Whole-transcriptome analysis with RNA-seq

Total RNA was extracted from 293MSR cells using the RNeasy Mini Kit (Qiagen Inc., Hilden, Germany). Library preparation was performed as previously described,^72^^,^^73^ and the quality and concentration of the libraries were assessed using an Agilent 2200 TapeStation (Agilent Technologies Inc.) with a D1000 ScreenTape assay. Libraries were then pooled at equal molecular concentrations and sequenced on an Illumina NextSeq 500 DNA sequencer using a 75-bp paired-end cycle sequencing kit (Illumina Inc., San Diego, CA). The sequencing data were trimmed and mapped to the human reference genome (GRCh38 release-95) using the CLC Genomics Workbench software (ver. 12.0.3; Qiagen Inc., RRID:SCR_011853). Mapped read counts were normalized to transcripts per million (TPM) and subsequently log_2_-transformed after adding 1.

##### Quantitative RT-PCR assay

Total RNA was extracted from 293MSR cells with or without the indicated siRNA transfection using ISOGEN II Reagent (NIPPON GENE, Tokyo, Japan) according to the manufacturer’s protocol. For siRNA-transfected cells, total RNA was collected 24 h after transfection. Reverse transcription (RT) was performed using 500 ng of total RNA with ReverTra Ace qPCR RT Master Mix (TOYOBO, Osaka, Japan). Quantitative RT-PCR (RT-qPCR) was performed using the LightCycler 480 System (Roche Diagnostics) with SYBR Advantage qPCR Premix (TOYOBO). mRNA expression levels were normalized to human GAPDH mRNA as an internal control and expressed as relative fold changes. PCR amplification was performed using a two-step protocol with the following conditions: pre-incubation at 95°C for 3 min, followed by 40 cycles of amplification at 95°C for 5 s and 60°C for 30 s. Melting curve analysis was then performed at 95°C for 5 min, 60°C for 60 s, and 97°C for the final melt. The primers used for quantitative RT-PCR are listed in the key resources table.

##### CFTR PM level measurement

The PM level of ΔF508-CFTR-Nluc(Ex) was measured as described previously.^32^ 293MSR WT or RNF5/185 DKO cells stably expressing ΔF508-CFTR-Nluc(Ex) under the control of the Tet-On system were transfected with the indicated siRNAs using Lipofectamine RNAiMAX (Thermo Fisher Scientific). The cells were then trypsinized, seeded into white 96-well plates, and treated with doxycycline (Dox; 1 μg/mL) for 2 days at 37°C to induce ΔF508-CFTR-Nluc(Ex) expression. Four days after siRNA transfection, cells were incubated with the cell-impermeable Extracellular NanoLuc® Substrate (Promega, CS313501, Custom Assay Services) in CO_2_-independent medium (Thermo Fisher Scientific; 50 μL/well) for 5–10 min. Luminescence was then measured using a Luminoskan (Thermo Fisher Scientific), Varioskan (Thermo Fisher Scientific), or EnSpire plate reader (PerkinElmer).

##### Western blotting

Western blotting was performed as previously described.^27^ Briefly, cells were solubilized in RIPA buffer supplemented with 1 mM PMSF (FUJIFILM Wako Pure Chemical Corporation), 5 μg/mL leupeptin (FUJIFILM Wako Pure Chemical Corporation), and 5 μg/mL pepstatin A (Peptide Institute, Inc.). Equal amounts of cell lysate proteins were subjected to SDS-PAGE, and the proteins were transferred onto nitrocellulose membranes using the Mini Trans-Blot cell system (Bio-Rad, Hercules, USA). Membranes were then blocked with 5% skim milk and incubated with primary antibodies, followed by HRP-conjugated secondary antibodies. Signals were detected using SuperSignal West Pico PLUS Chemiluminescent Substrate (Thermo Fisher Scientific), ImmunoStar Zeta (FUJIFILM Wako Pure Chemical Corporation), or ImmunoStar LD (FUJIFILM Wako Pure Chemical Corporation). Western blot images were acquired using either the LAS 4000 mini (Cytiva Life Sciences, Marlborough, MA, USA) or the FUSION Chemiluminescence Imaging System (Vilber Bio Imaging, Quantum, France). In some experiments, the HiBiT tag was detected using the Nano-Glo® HiBiT Blotting System (Promega), following the manufacturer’s instructions. As a loading control, total proteins were stained with Ponceau S. Densitometric analysis of Western blots was performed using ImageQuant (LAS 4000 mini software, Cytiva) or Evolution-Capt (Fusion software, Vilber Bio Imaging).

##### CFTR ubiquitination measurement by western blotting

CFTR ubiquitination in cells was measured as described previously.^27^ Briefly, 293MSR WT and RNF5/185 DKO cells stably expressing HBH-ΔF508-CFTR-3HA were transfected with the indicated siRNAs at a final concentration of 10 nM. Four days after transfection, cells were treated with MG-132 (10 μM) for 3 h at 37°C and then lysed in RIPA buffer containing leupeptin (5 μg/mL), pepstatin A (5 μg/mL), PMSF (1 mM), MG-132 (10 μM), and N-ethylmaleimide (NEM; 5 mM). Cell lysates were incubated with NeutrAvidin agarose beads (Thermo Fisher Scientific) overnight at 4°C to isolate HBH-ΔF508-CFTR. The bead-bound CFTR complex was incubated with 1% SDS in RIPA buffer for 5 min at room temperature to denature CFTR, followed by five washes with RIPA buffer containing 2 M urea. CFTR was eluted in urea elution buffer containing 8 M urea, 2% SDS, and 3 mM biotin for 30 min at room temperature and analyzed by Western blotting using anti-Ub (P4D1) and anti-HA (16B12; BioLegend) antibodies. CFTR ubiquitination levels were quantified by densitometry and normalized to the amount of CFTR in the precipitate.

##### Ub ELISA

CFTR ubiquitination levels in 293MSR and RNF5/185 DKO cells were measured as described previously.^36^^,^^74^ Briefly, 293MSR WT and RNF5/185 DKO cells stably expressing HBH-ΔF508-CFTR-3HA were transfected with the indicated siRNAs at a final concentration of 10 nM. Four days after transfection, cells were treated with MG-132 (10 μM) for 3 h at 37°C and then lysed in RIPA buffer containing leupeptin (5 μg/mL), pepstatin A (5 μg/mL), PMSF (1 mM), MG-132 (10 μM), and N-ethylmaleimide (NEM; 5 mM). HBH-ΔF508-CFTR-3HA in the cell lysates was immobilized on NeutrAvidin-coated white 96-well plates and denatured in 8 M urea for 5 min at room temperature. The plates were then washed five times with 2 M urea in RIPA buffer, followed by four washes with 0.1% NP-40 in PBS. After blocking with 0.1% BSA, ubiquitination and CFTR levels were detected using anti-K48-linked Ub (Apu2; Merck Millipore) and anti-HA (16B12; BioLegend) antibodies, respectively. CFTR ubiquitination levels were normalized to the total CFTR levels.

To detect Myc-tagged Ub conjugation on CFTR, 293MSR WT and RNF5/185 DKO HBH-ΔF508-CFTR-3HA cells were transfected with Myc-Ub WT or Ub variants containing only a single lysine (K) residue at the specified position (K11, K29, K33, K48, or K63). Two days post-transfection, cells were treated with 10 μM MG-132 for 3 h at 37°C to inhibit proteasomal degradation. Myc-Ub levels on CFTR were detected using ELISA, as described above, with an anti-Myc antibody (9E10, FUJIFILM Wako Pure Chemical Corporation). CFTR ubiquitination levels were then normalized to total CFTR levels, which were measured using an anti-HA antibody.

##### Pull-down experiment

To examine the interaction between AMFR and CFTR domains, 293MSR RNF5/185 DKO cells were transfected with MB-AMFR along with CFTR NBD1-HiBiT, MSD1-HiBiT, or MSD2-HiBiT. Two days post-transfection, cells were treated with 10 μM MG-132 for 3 h at 37°C and then solubilized in mild lysis buffer as described above. The cell lysates were incubated with NeutrAvidin agarose beads (Thermo Fisher Scientific) for 2 h at 4°C. After four washes with mild lysis buffer, the protein complexes were eluted in urea elution buffer (8 M urea, 2% SDS, 3 mM biotin) at room temperature for 30 min and analyzed by Western blotting, as shown in the figures.

##### HiBiT and Nluc degradation assay

The HiBiT and Nluc degradation assays were performed as previously described.^27^^,^^33^ Briefly, proteins fused with HiBiT tag in the cytoplasmic C-terminal region (CT) and cytosolic LgBiT (pBiT1.1-N [TK/LgBiT], Promega) were transiently expressed in 293MSR and RNF5/185 DKO cells transfected with the siRNAs indicated in the figure legends. TMUB1-HiBiT and TMUB2-HiBiT were expressed using the pLIX402 Gateway cloning vector and 1 μg/mL Dox treatment for 24 h. Four days post-transfection, cells seeded in 96-well plates were incubated with 50 μL of 0.1x Nano-Glo® Endurazine (Promega) in CO_2_-independent medium for 2.5 h at 37°C in 5% CO_2_. For the Nluc degradation assay, subconfluent BEAS-2B cells stably expressing Dox-inducible ΔF508-CFTR-Nluc(CT) or Insig-1-Nluc(CT) were transfected with siRNA, and the expression of ΔF508-CFTR-Nluc(CT) was induced by treating the cells with 1 μg/mL Dox for 2 days. To measure the degradation kinetics, CHX was added to a final concentration of 100 μg/mL, and luminescence was measured continuously at 5-min intervals for 3–12 h using a Luminoskan plate reader (Thermo Fisher Scientific).

For the inhibitor assay, cells were incubated with BTZ (100 nM) or BafA1 (1 μM) during the 2.5-h Nano-Glo® Endurazine incubation, and the inhibitors were maintained during the CHX chase. The luminescence signal of CHX-treated cells was normalized to the signal of untreated cells, and the degradation rate was calculated by fitting the luminescence decay to a one-phase exponential decay function using GraphPad Prism 8 (GraphPad Software, La Jolla, CA, USA) as described previously.^27^

##### HiBiT cytosolic-accessibility and ER-disappearance assays

The cytosolic-accessibility and ER-disappearance assays were performed as previously described.^27^ Briefly, ΔF508-CFTR carrying HiBiT in an ER-luminal/extracellular loop was coexpressed with cytosolic LgBiT for the cytosolic-accessibility assay or ER-luminal LgBiT (CNXss-LgBiT-KDEL) for the ER-disappearance assay in 293MSR WT and RNF5/185 DKO cells transfected with the indicated siRNAs. Four days after transfection, cells seeded in 96-well plates were incubated with 50 μL of 0.5× Nano-Glo® Endurazine (Promega) for the cytosolic-accessibility assay or 0.1× Nano-Glo® Endurazine for the ER-disappearance assay, in CO_2_-independent medium for 2.5 h at 37°C in 5% CO_2_. To monitor cytosolic accessibility associated with CFTR extraction, 10 μL of 60 μM MG-132 (final concentration 10 μM) in CO_2_-independent medium was added to each well. Luminescence was measured continuously at 5-min intervals for 3 h using a Luminoskan plate reader (Thermo Fisher Scientific). Signal generated by complementation of the ER-luminal HiBiT tag with cytosolic LgBiT was used as an operational measure of cytosolic accessibility associated with CFTR extraction. The increase in signal after MG-132 addition was used to calculate the apparent retrotranslocation-associated rate by linear fitting. For the ER-disappearance assay, CHX was added to a final concentration of 100 μg/mL and luminescence was monitored at 5-min intervals for 3 h using the Luminoskan plate reader (Thermo Fisher Scientific). The signal from CHX-treated cells was normalized to untreated cells, and the ER-disappearance rate was calculated by fitting the decay to a one-phase exponential function using GraphPad Prism 8.

##### Structural representations

Structural models of the untagged WT CFTR–RNF5–RNF185 and untagged WT CFTR–AMFR complexes (Figure S4) were predicted using AlphaFold 3 (https://alphafoldserver.com) based on the amino acid sequences of CFTR (UniProt: P13569), RNF5 (UniProt: Q99942), RNF185 (UniProt: Q96GF1), and AMFR (UniProt: Q9UKV5). Structural representations were generated using PyMOL (version 3.0.0; Schrödinger, LLC). Putative interacting residues within the CFTR transmembrane (TM) region were identified as those located within 5 Å of RNF5, RNF185, or AMFR in the respective predicted complex structures.

### Quantification and statistical analysis

Except for RNA-seq analysis (*n* = 1) and selected qRT-PCR experiments performed to validate gene knockdown (*n* = 2), quantitative data were obtained from at least three independent experiments. Data are presented as the mean ± standard deviation (SD), unless otherwise indicated. The value of n represents the number of biological replicates unless otherwise stated. The sample sizes (n), statistical tests, and multiple-comparison procedures used for individual experiments are detailed in the corresponding figure legends. Statistical analyses were performed using GraphPad Prism 8. Statistical significance was assessed using two-tailed paired Student’s t-tests, two-tailed Welch’s t-tests, one-way or two-way ANOVA, or repeated-measures ANOVA, followed by Dunnett’s or Holm–Sidak multiple-comparison tests, as appropriate. A *p* value of less than 0.05 was considered statistically significant. Statistical significance is indicated as follows: ∗*p* < 0.05, ∗∗*p* < 0.01, ∗∗∗*p* < 0.001, and ∗∗∗∗*p* < 0.0001; ns, not significant. Data were assumed to be normally distributed, although normality was not formally tested.
