# Supplementary material for: RNF5/185 deficiency reveals a distinct compensatory role for AMFR in mutant CFTR ERAD
Source: iScience. 2026 Nov 10;29(11):118195. doi: 10.1016/j.isci.2026.118195 (PMC13770712; doi:10.1016/j.isci.2026.118195)
Supplement: Supplementary file 1 [file mmc1.pdf]

iScience, Volume *29*

Supplemental information

RNF5/185 deficiency reveals a distinct compensatory role for AMFR in mutant CFTR ERAD

Uta Nakayamada, Aika Fujii, Saori Kato, Yuki Karasaki, Mayuko Nakatani, Momone Yamamoto, Natsuki Manai, Yuka Kamada, and Tsukasa Okiyoneda

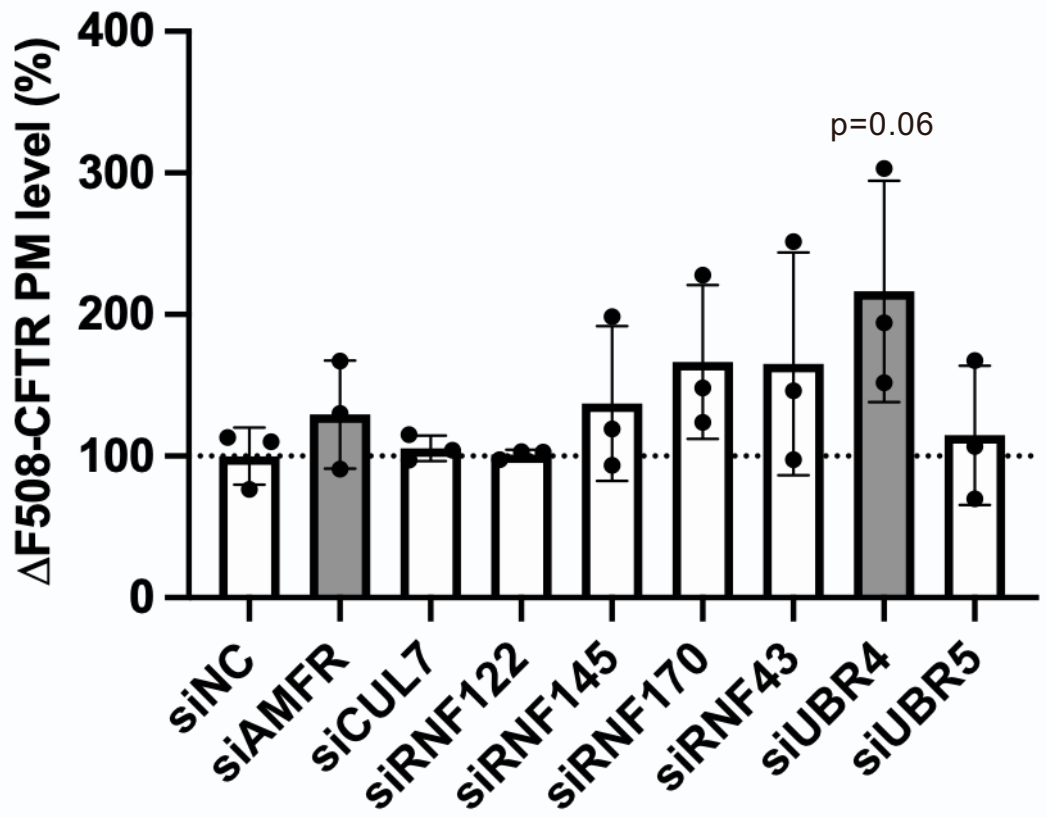

Figure S1.

Effects of selected E3 ligase KD on cell surface ΔF508-CFTR–Nluc(Ex) in 293MSR WT cells

The assay was performed as described in Fig. 1B, following transfection with 10 nM non-targeting control siRNA (siNC) or pooled siRNAs (n = 3). Statistical significance was assessed using one-way ANOVA with Dunnett's multiple comparisons test. Data are presented as mean ± SD.

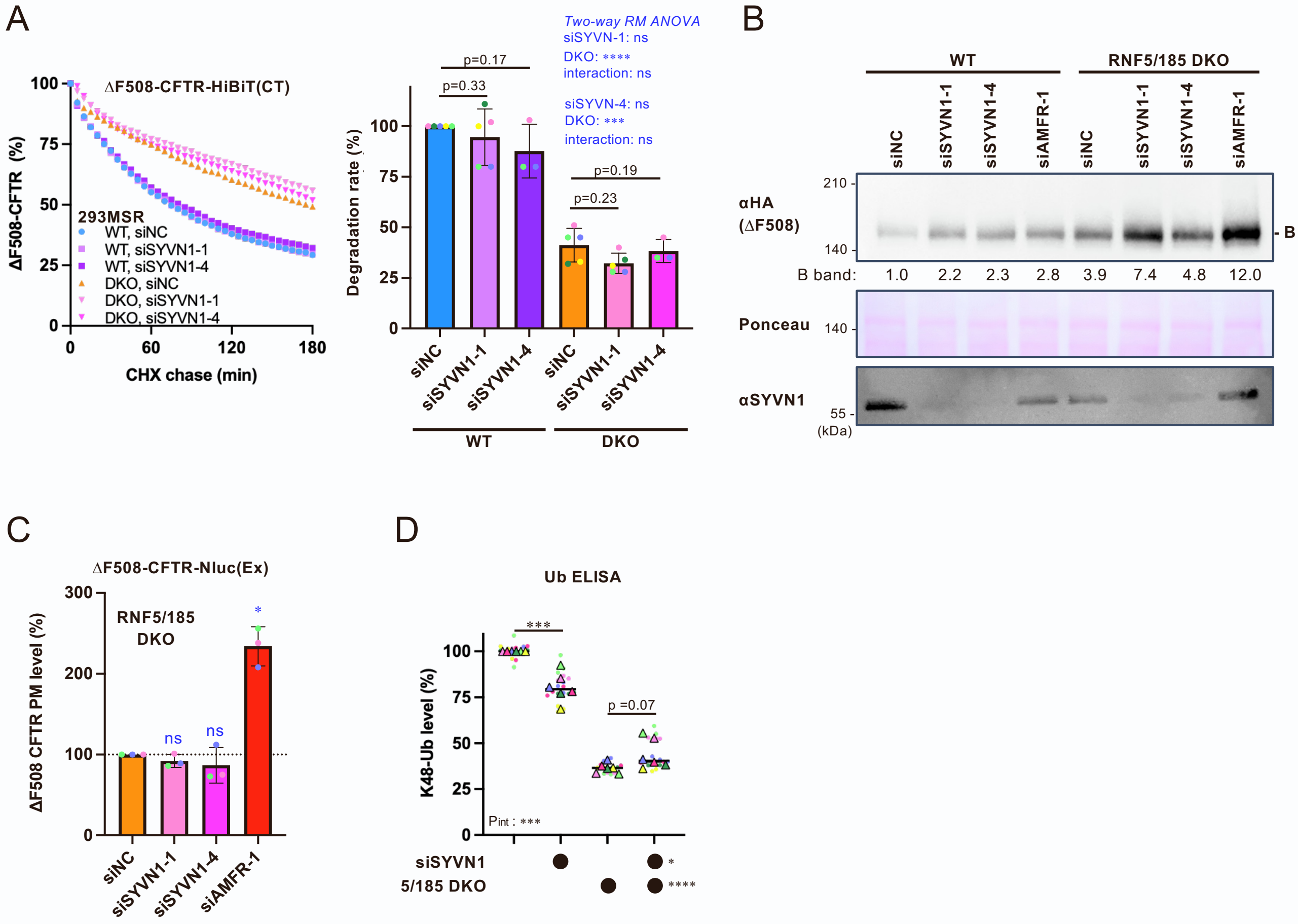

Figure S2.

Limited role of SYVN1 in compensatory CFTR ERAD in RNF5/185 DKO cells

(A) Kinetic degradation of ΔF508-CFTR-HiBiT(CT) in 293MSR WT and RNF5/185 DKO cells transfected with 10 nM siNC, siSYVN1-1, or siSYVN1-4 was assessed as described in Fig. 2A. The ERAD rate was calculated by fitting the initial degradation portion of each kinetic degradation curve (right, n = 3-5). Each biological replicate (n) is color-coded, with the averages from three to four technical replicates represented by triangles. Statistical significance was determined using two-way RM ANOVA with Holm–Sidak multiple comparison tests.

(B) Western blot analysis of HBH-ΔF508-CFTR-3HA levels in 293MSR WT and RNF5/185 DKO cells transfected as in panel A. Ponceau S staining was used as a loading control. The relative levels of immature ΔF508-CFTR (band B) were quantified via densitometry and are indicated below the anti-HA blot. SYVN1 KD was confirmed using an anti-SYVN1 antibody.

(C) PM expression levels of ΔF508-CFTR-Nluc(Ex) in RNF5/185 DKO cells transfected with 10 nM of the indicated siRNAs (n = 3). Statistical significance was evaluated using one-way RM ANOVA with Dunnett's multiple comparison tests.

(D) K48-linked polyubiquitination levels of ΔF508-CFTR in HBH-ΔF508-CFTR-3HA 293MSR WT and RNF5/185 DKO cells transfected with 10 nM siNC or pooled siSYVN1 (siSYVN1-1 and siSYVN1-4) were quantified by Ub ELISA using an anti-K48-Ub antibody. Cells were treated with 10 μM MG-132 for 3 hours before lysis. Ub levels were normalized to CFTR levels, quantified by ELISA using an anti-HA antibody (n = 6). Statistical significance was assessed using two-way RM ANOVA with Holm-Sidak multiple comparison tests. Each biological replicate (n) is color-coded.

Data are presented as mean ± SD. ∗P < 0.05, ∗∗∗P < 0.001, ∗∗∗∗P < 0.0001, ns (not significant).

Figure S3

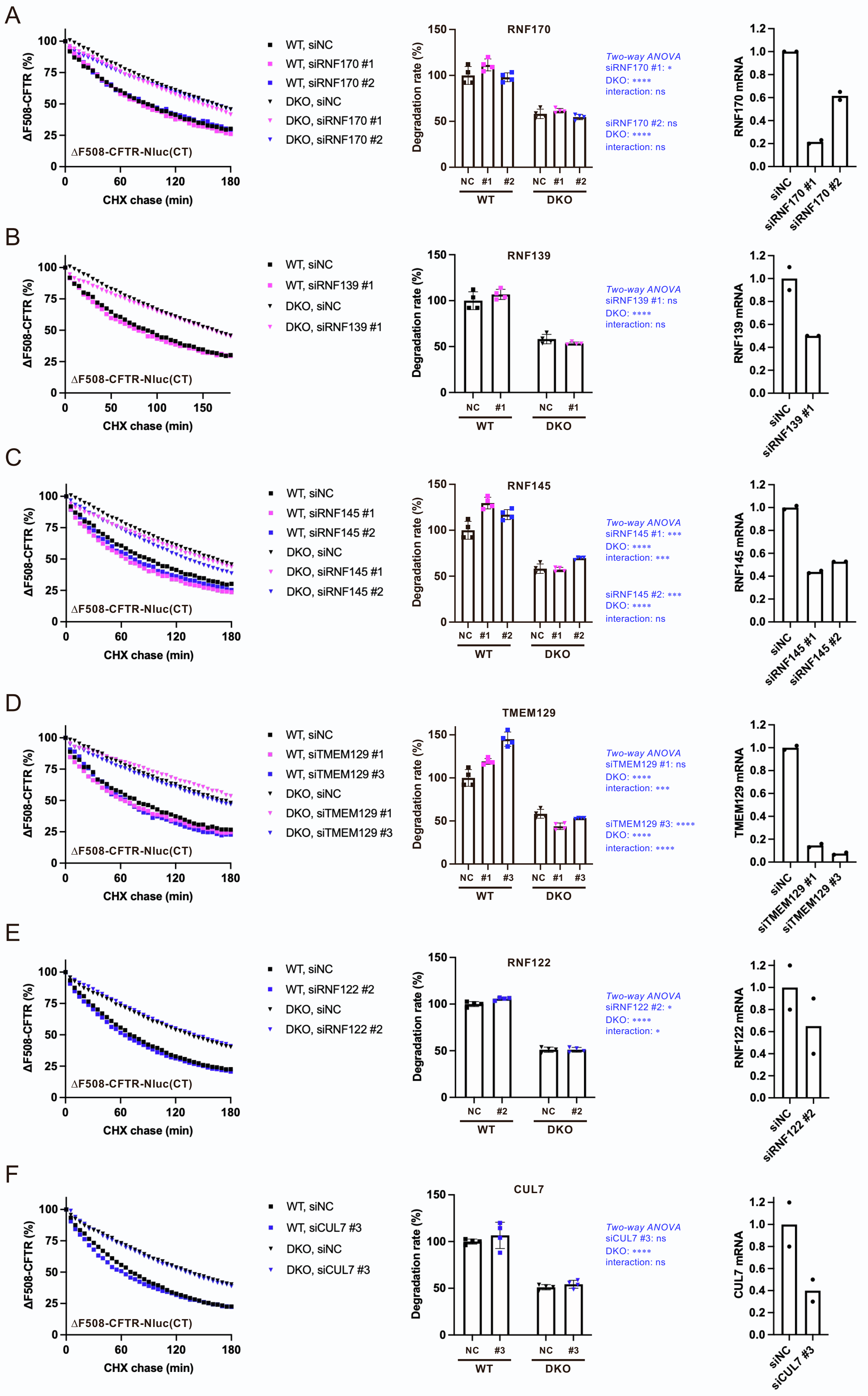

A
ΔF508-CFTR (%)
ΔF508-CFTR-Nluc(CT)
CHX chase (min)
WT, siNC
WT, siRNF170 #1
WT, siRNF170 #2
DKO, siNC
DKO, siRNF170 #1
DKO, siRNF170 #2
RNF170
Degradation rate (%)
NC #1 #2 NC #1 #2
WT DKO
Two-way ANOVA
siRNF170 #1: *
DKO: ****
interaction: ns
siRNF170 #2: ns
DKO: ****
interaction: ns
RNF170 mRNA
siNC
siRNF170 #1
siRNF170 #2
B
ΔF508-CFTR (%)
ΔF508-CFTR-Nluc(CT)
CHX chase (min)
WT, siNC
WT, siRNF139 #1
DKO, siNC
DKO, siRNF139 #1
RNF139
Degradation rate (%)
NC #1 NC #1
WT DKO
Two-way ANOVA
siRNF139 #1: ns
DKO: ****
interaction: ns
RNF139 mRNA
siNC
siRNF139 #1
C
ΔF508-CFTR (%)
ΔF508-CFTR-Nluc(CT)
CHX chase (min)
WT, siNC
WT, siRNF145 #1
WT, siRNF145 #2
DKO, siNC
DKO, siRNF145 #1
DKO, siRNF145 #2
RNF145
Degradation rate (%)
NC #1 #2 NC #1 #2
WT DKO
Two-way ANOVA
siRNF145 #1: ***
DKO: ****
interaction: ***
siRNF145 #2: ***
DKO: ****
interaction: ns
RNF145 mRNA
siNC
siRNF145 #1
siRNF145 #2
D
ΔF508-CFTR (%)
ΔF508-CFTR-Nluc(CT)
CHX chase (min)
WT, siNC
WT, siTMEM129 #1
WT, siTMEM129 #3
DKO, siNC
DKO, siTMEM129 #1
DKO, siTMEM129 #3
TMEM129
Degradation rate (%)
NC #1 #3 NC #1 #3
WT DKO
Two-way ANOVA
siTMEM129 #1: ns
DKO: ****
interaction: ***
siTMEM129 #3: ****
DKO: ****
interaction: ****
TMEM129 mRNA
siNC
siTMEM129 #1
siTMEM129 #3
E
ΔF508-CFTR (%)
ΔF508-CFTR-Nluc(CT)
CHX chase (min)
WT, siNC
WT, siRNF122 #2
DKO, siNC
DKO, siRNF122 #2
RNF122
Degradation rate (%)
NC #2 NC #2
WT DKO
Two-way ANOVA
siRNF122 #2: *
DKO: ****
interaction: *
RNF122 mRNA
siNC
siRNF122 #2
F
ΔF508-CFTR (%)
ΔF508-CFTR-Nluc(CT)
CHX chase (min)
WT, siNC
WT, siCUL7 #3
DKO, siNC
DKO, siCUL7 #3
CUL7
Degradation rate (%)
NC #3 NC #3
WT DKO
Two-way ANOVA
siCUL7 #3: ns
DKO: ****
interaction: ns
CUL7 mRNA
siNC
siCUL7 #3

Figure S3-continued

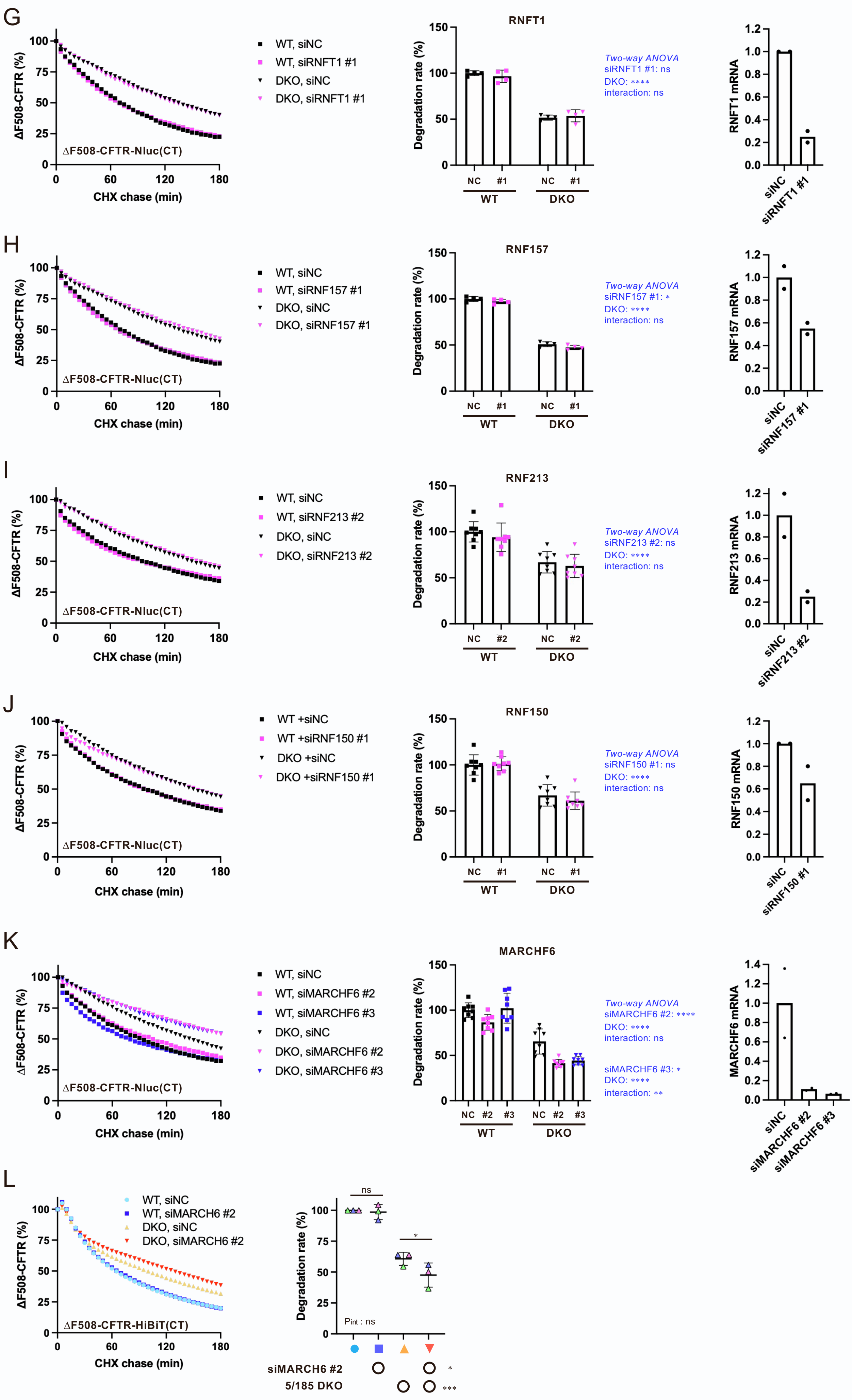

Figure S3.

Effects of KD of ER-associated E3 ligases on CFTR ERAD in WT and RNF5/185 DKO cells

(A–K) Kinetic degradation of ΔF508-CFTR-NLuc(CT) in 293MSR WT and RNF5/185 DKO cells transfected with the indicated siRNAs (10 nM) was assessed as described in Fig. 2A. ERAD rates were determined by fitting the initial phase of the degradation curves (n = 4 – 8). Knockdown of each target gene was confirmed by qRT-PCR (right panels, n = 2).

(L) Kinetic degradation of ΔF508-CFTR-HiBiT(CT) in 293MSR WT and RNF5/185 DKO cells transfected with the indicated siMARCHF6 was assessed as described in Fig. 2A. ERAD rates were determined by fitting the initial phase of the degradation curves (right; n = 3). Each biological replicate is color-coded, and the mean of three to four technical replicates is shown as triangles.

Statistical significance was assessed using two-way ANOVA (A-K) or two-way repeated-measures (RM) ANOVA (L) followed by Holm–Sidak's multiple comparisons test. Data are presented as mean ± SD. ∗P < 0.05, ∗∗P < 0.01, ∗∗∗P < 0.001, ∗∗∗∗P < 0.0001; ns, not significant.

A

CFTR-RNF5-RNF185 complex AF model 0

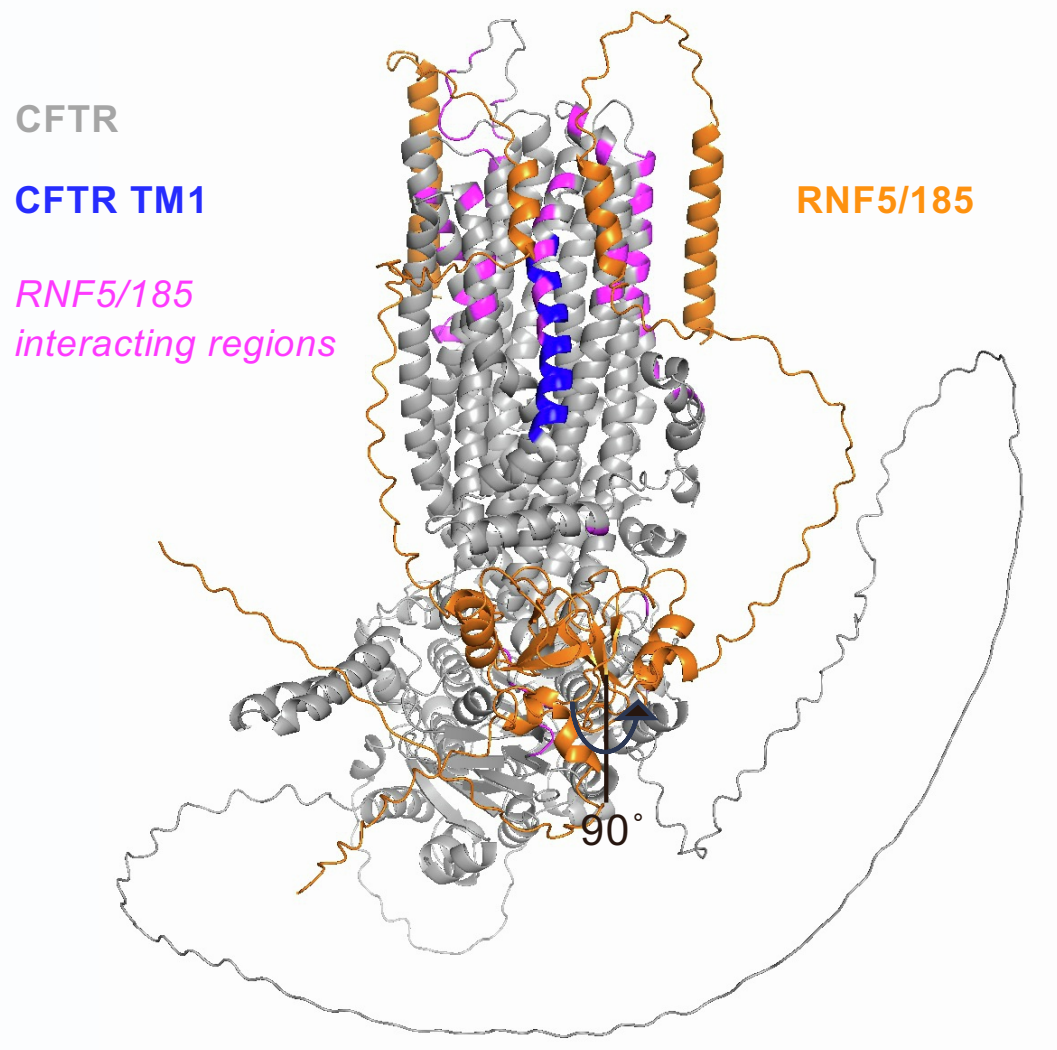

CFTR-RNF5-RNF185 complex

	AF model	CFTR TM1 (78-98 aa)
RNF5/185	0	FWRFMFYGIFLYLGEVTKAVQ
	1	none
	2	FWRFMFYGIFLYLGEVTKAVQ
	3	none
	4	FWRFMFYGIFLYLGEVTKAVQ

RNF5/185 interacting residues

B

CFTR-AMFR complex AF model 0

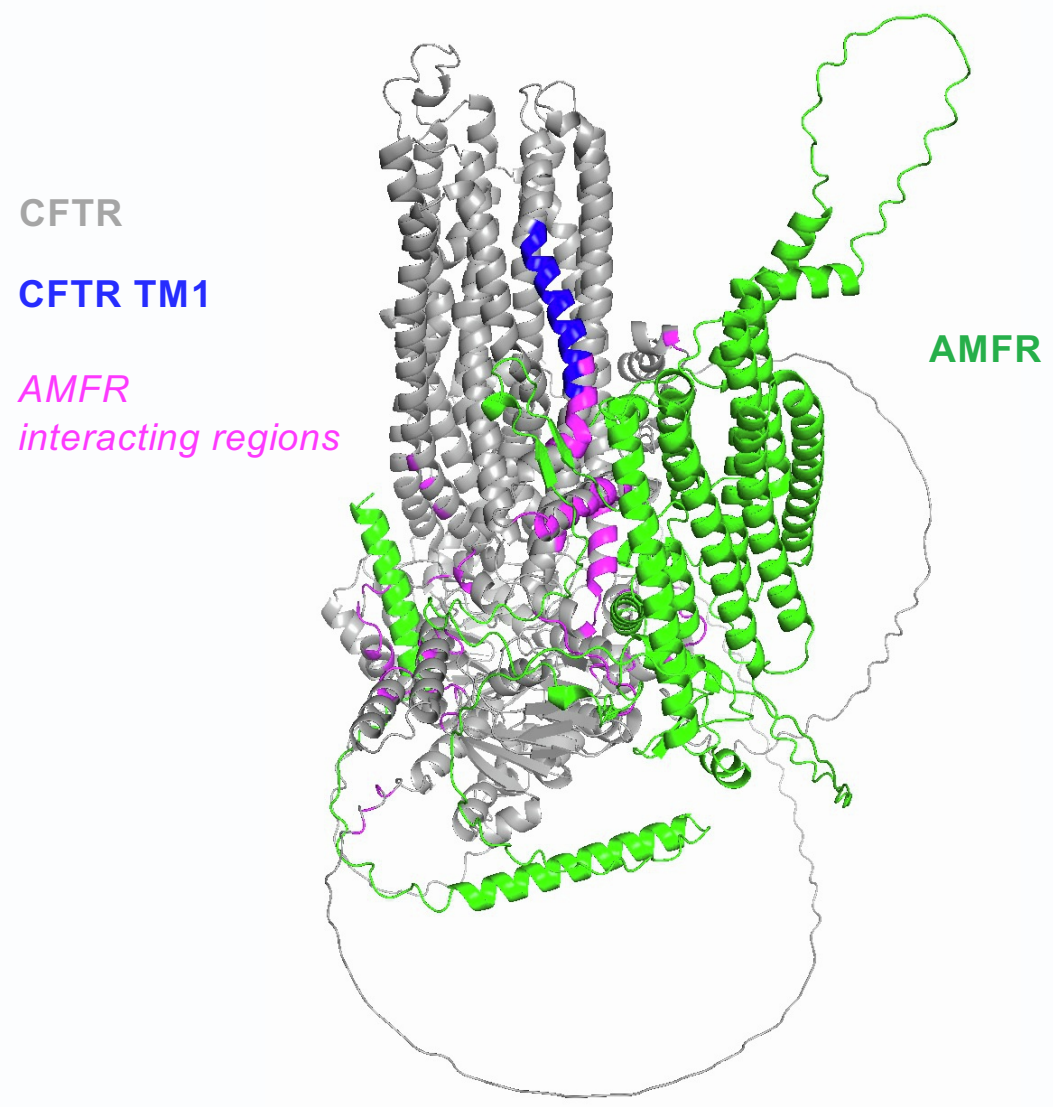

CFTR-AMFR complex

	AF model	CFTR TM1 (78-98 aa)
AMFR	0	FWRFMFYGIFLYLGEVTKAVQ
	1	FWRFMFYGIFLYLGEVTKAVQ
	2	FWRFMFYGIFLYLGEVTKAVQ
	3	FWRFMFYGIFLYLGEVTKAVQ
	4	FWRFMFYGIFLYLGEVTKAVQ

AMFR interacting residues

Figure S4.

Predicted structures of CFTR-RNF5-RNF185 and CFTR-AMFR complexes

(A) Structural model of the CFTR (gray)-RNF5/185 (orange) complex predicted using AlphaFold3 (AF3).

(B) Structural model of the CFTR (gray)-AMFR (green) complex predicted as in panel A.

The transmembrane domain 1 (TM1) of CFTR (blue) is highlighted, and putative interaction sites with RNF5/185 (A) and AMFR (B) are shown in magenta. Residues in the CFTR TM1 region predicted to interact with RNF5/185 or AMFR are listed based on five independent AF3 models for each complex.

Data S1: Original, uncropped Western blot images corresponding to the immunoblot analyses presented in the main and supplemental figures.

Figure 1

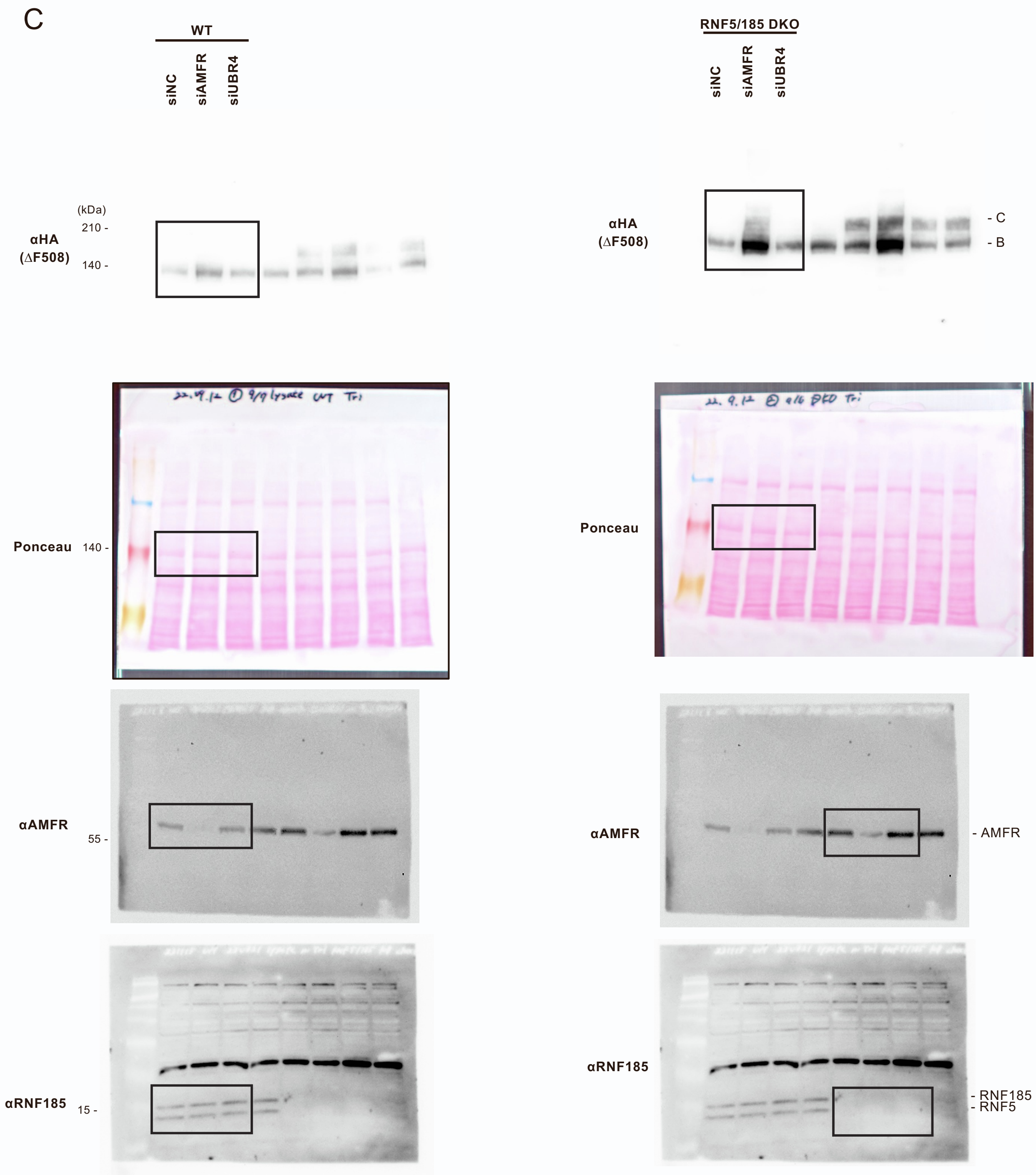

Figure 1

E

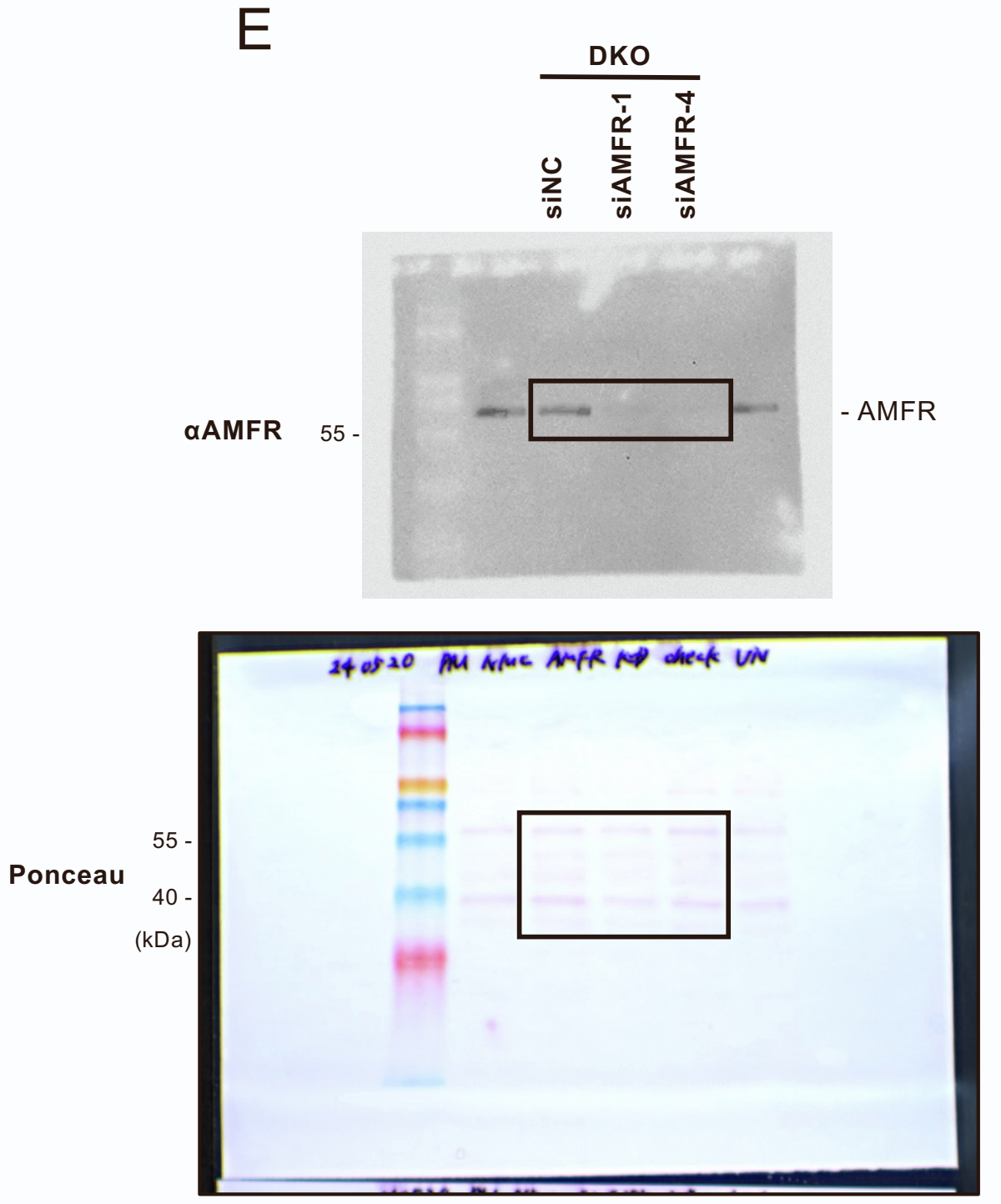
DKO
siNC
siAMFR-1
siAMFR-4
αAMFR
55 -
- AMFR
Ponceau
55 -
40 -
(kDa)

G

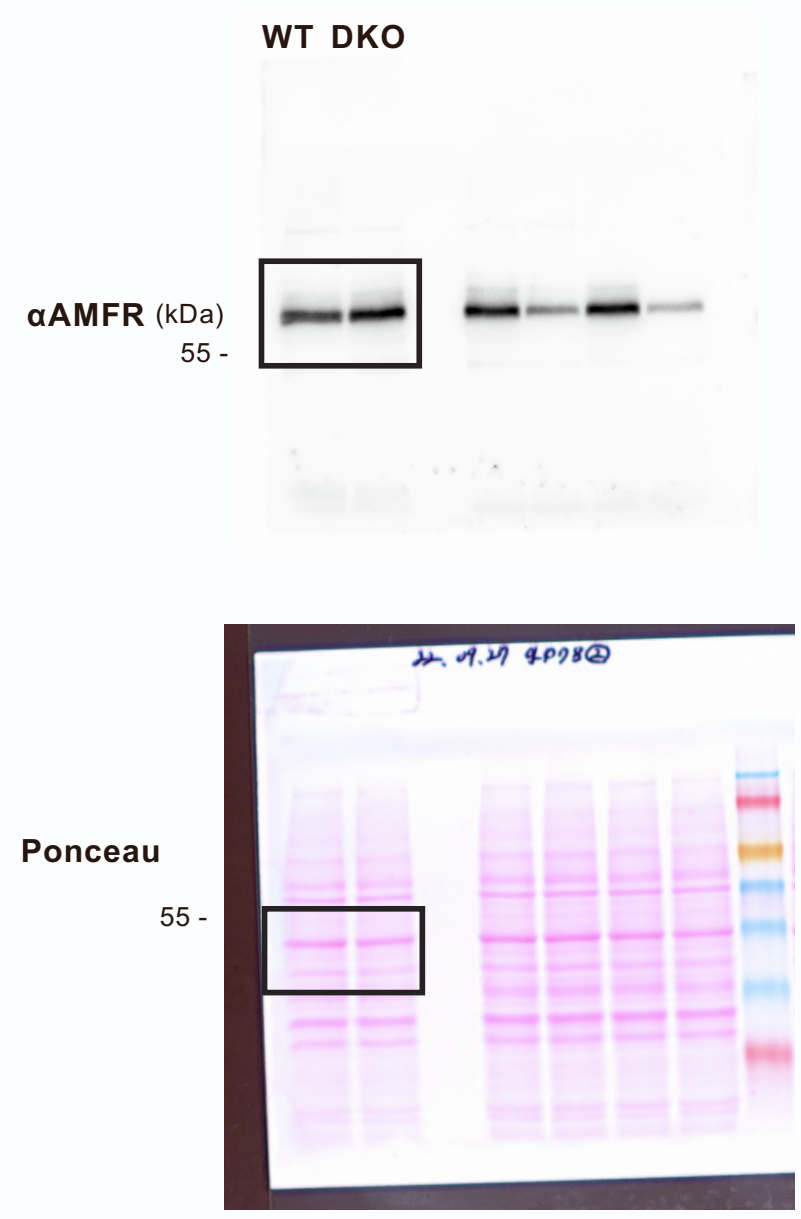
WT DKO
αAMFR (kDa)
55 -
Ponceau
55 -

H

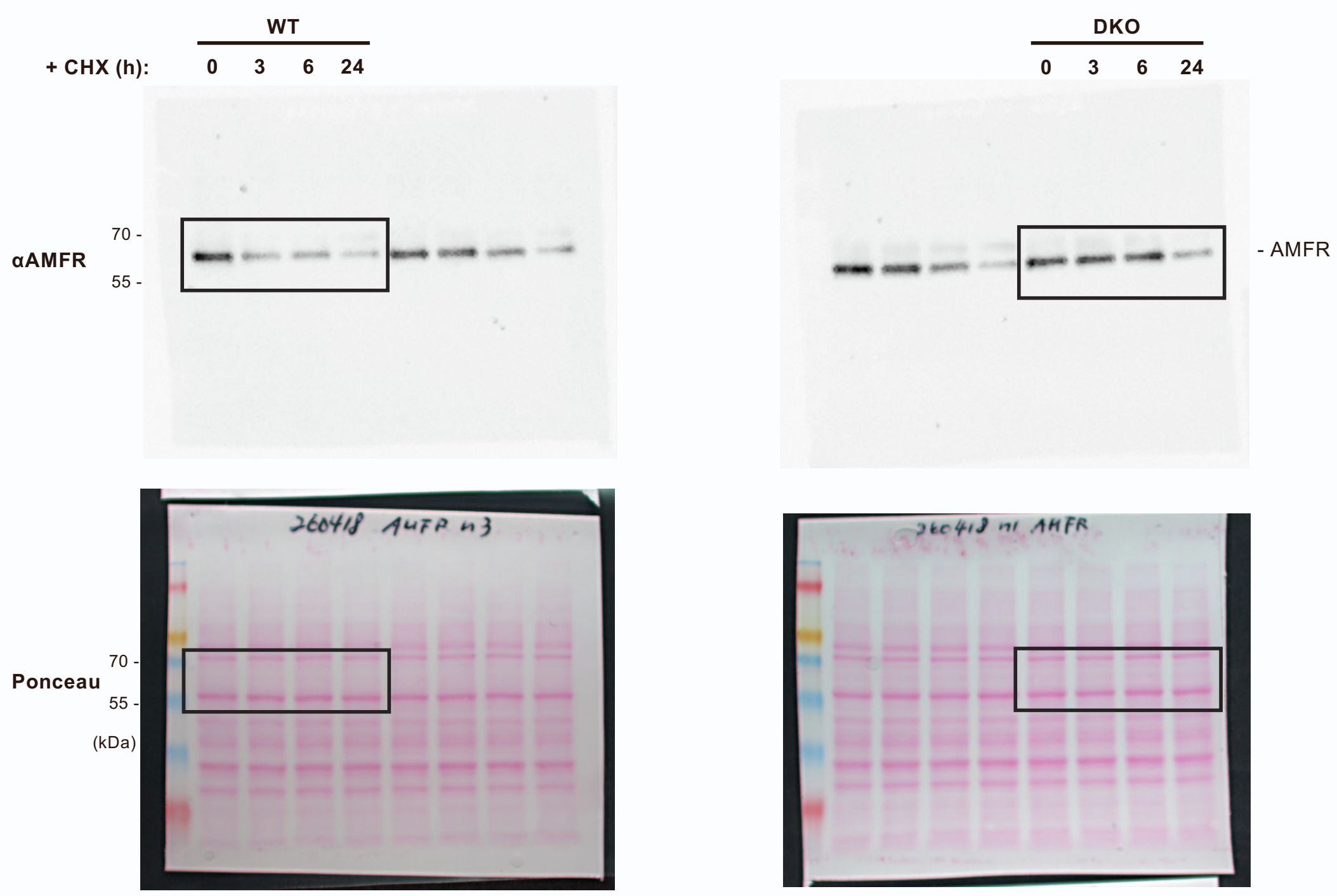
WT
DKO
+ CHX (h):
0 3 6 24
0 3 6 24
αAMFR
70 -
55 -
- AMFR
Ponceau
70 -
55 -
(kDa)

Figure 2

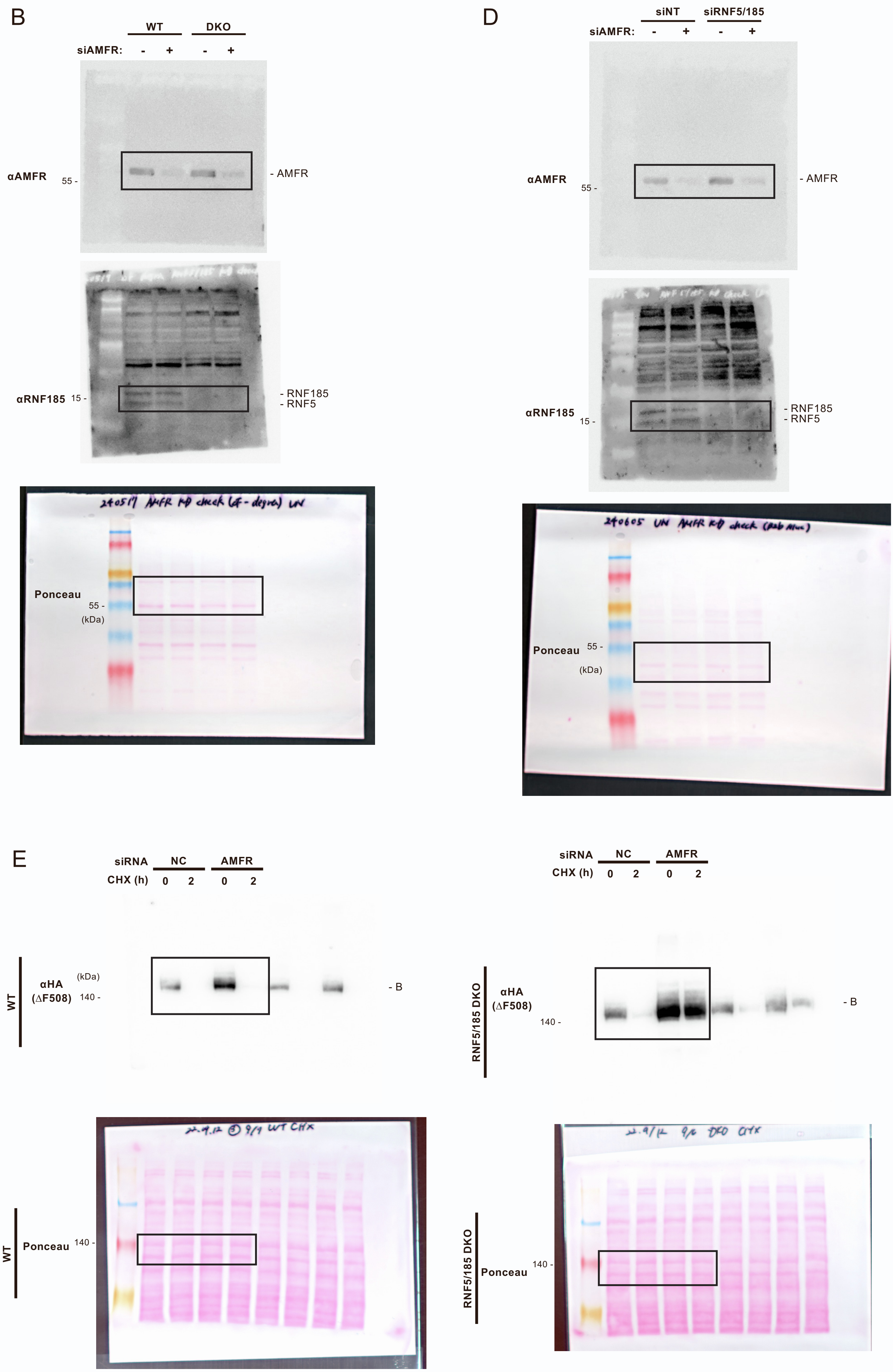
B
WT
DKO
siAMFR: - + - +
αAMFR
55 -
- AMFR
αRNF185
15 -
- RNF185
- RNF5
Ponceau
55 -
(kDa)
D
siNT
siRNF5/185
siAMFR: - + - +
αAMFR
55 -
- AMFR
αRNF185
15 -
- RNF185
- RNF5
Ponceau
55 -
(kDa)
E
siRNA NC AMFR
CHX (h) 0 2 0 2
WT
αHA
(ΔF508)
(kDa)
140 -
- B
RNF5/185 DKO
αHA
(ΔF508)
140 -
- B
WT
Ponceau
140 -
RNF5/185 DKO
Ponceau
140 -

Figure 4

A

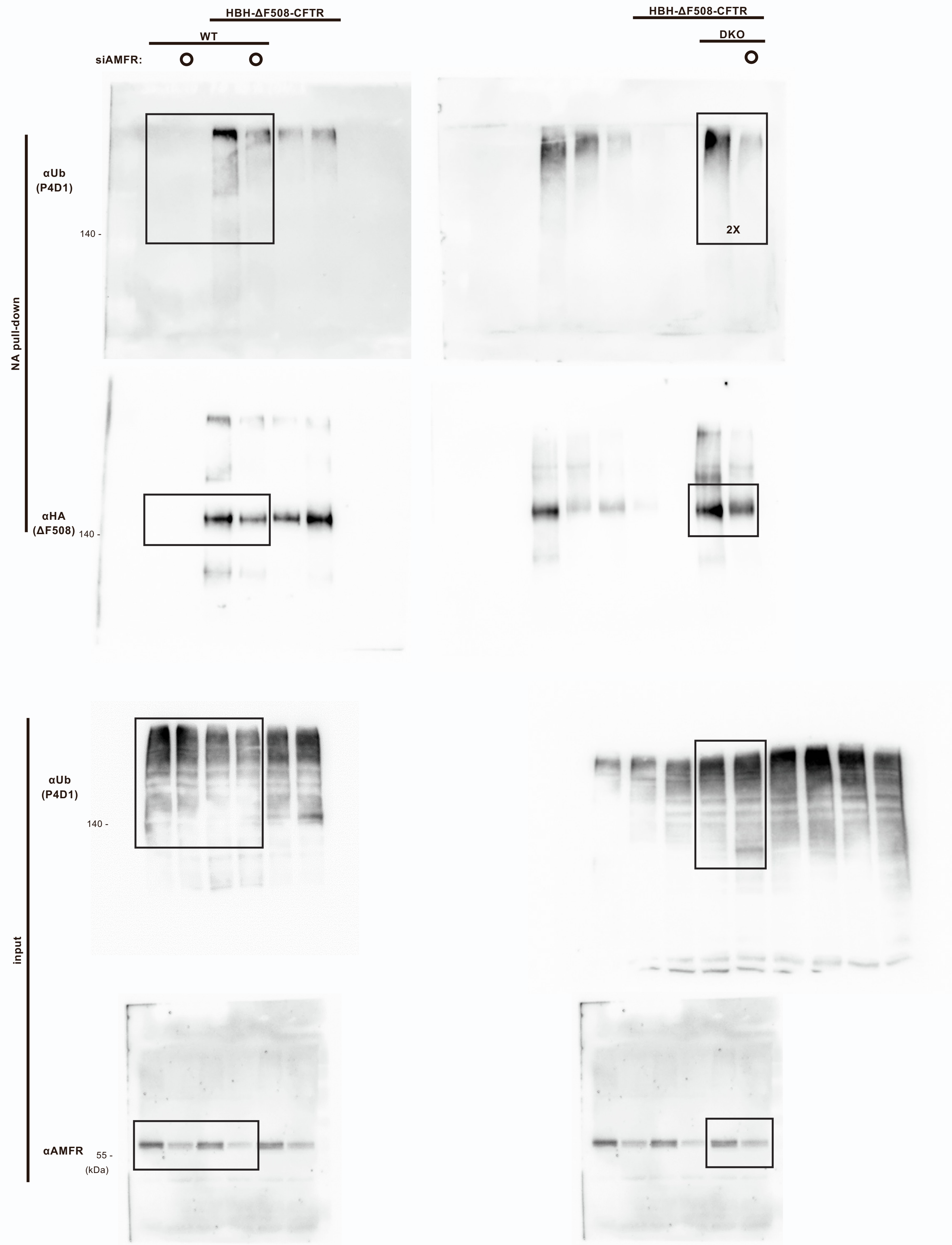

HBH-ΔF508-CFTR
WT
siAMFR:
HBH-ΔF508-CFTR
DKO
αUb
(P4D1)
140 -
2X
NA pull-down
αHA
(ΔF508)
140 -
input
αUb
(P4D1)
140 -
αAMFR
55 -
(kDa)

Figure 5

D

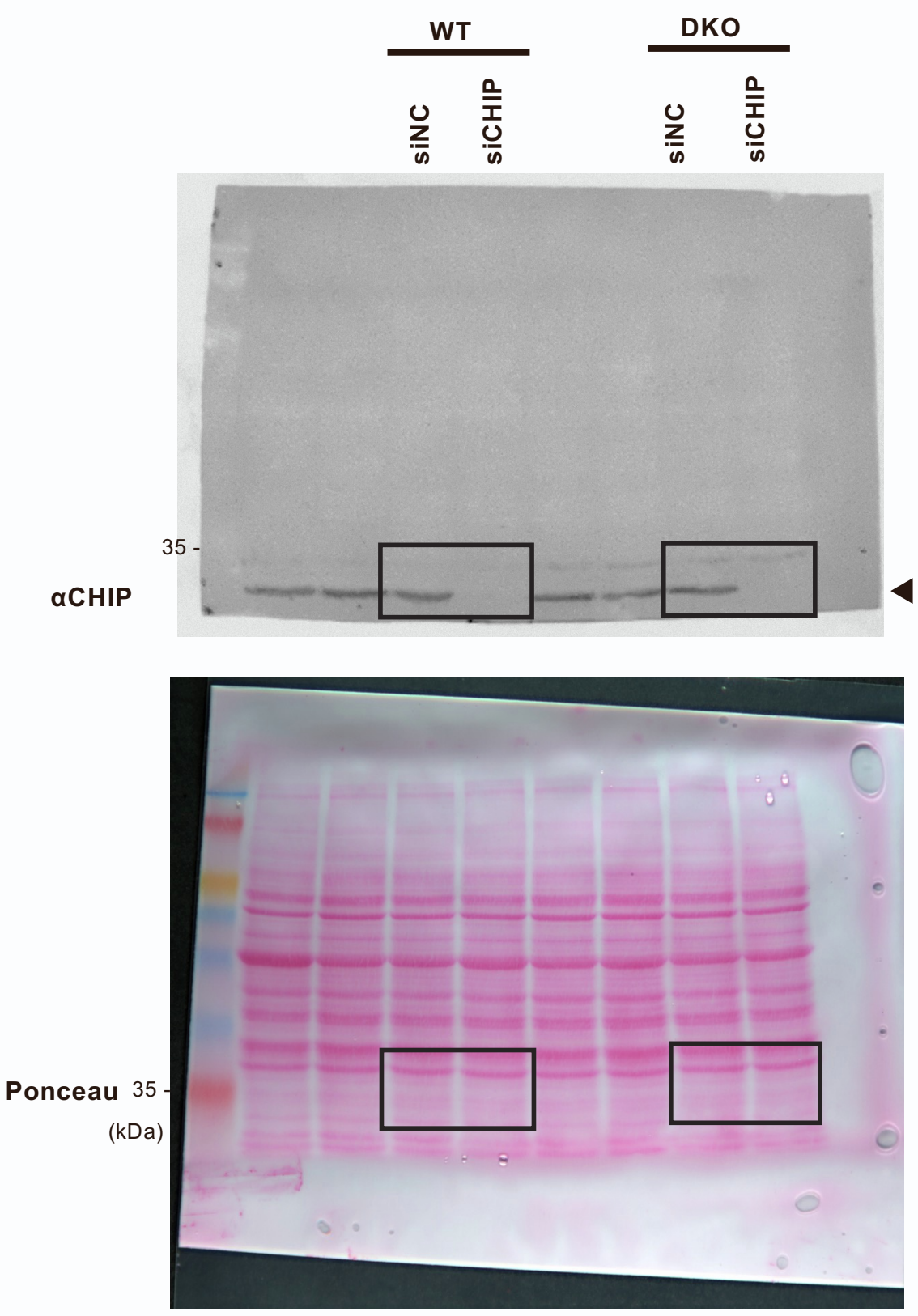

WT
DKO
siNC
siCHIP
siNC
siCHIP
35 -
αCHIP
Ponceau
35 -
(kDa)

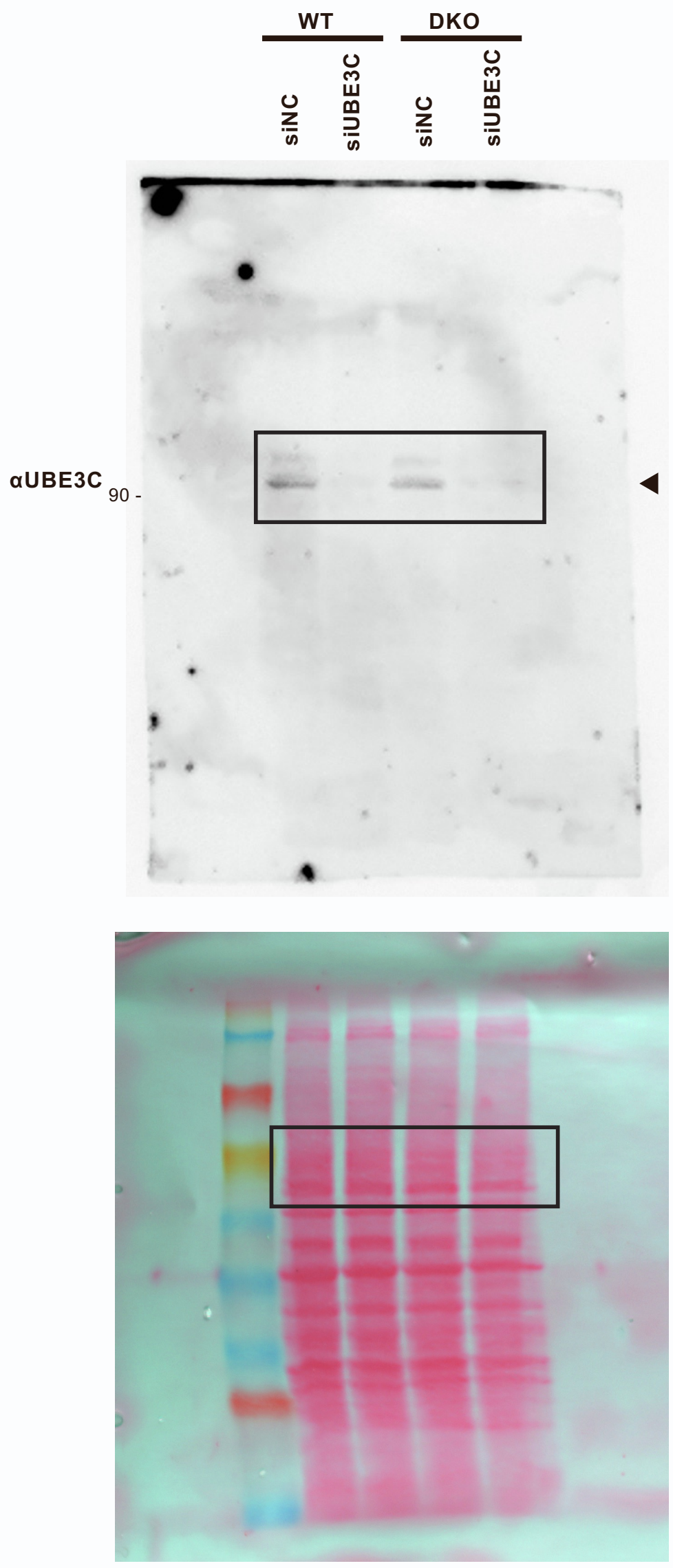

WT
DKO
siNC
siUBE3C
siNC
siUBE3C
αUBE3C
90 -

Figure 6

I

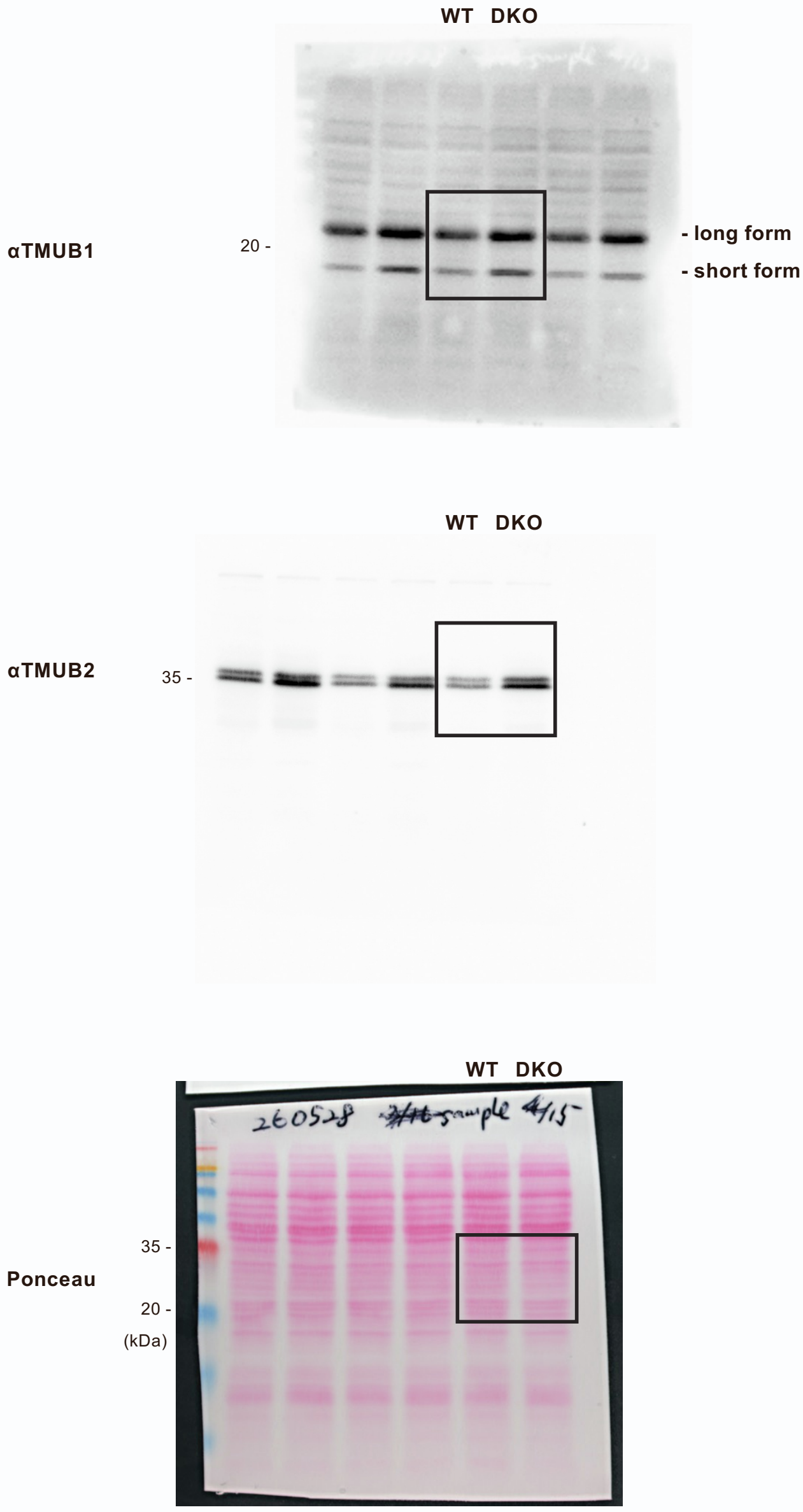
WT DKO
αTMUB1
20 -
- long form
- short form
WT DKO
αTMUB2
35 -
WT DKO
Ponceau
35 -
20 -
(kDa)

Figure 7

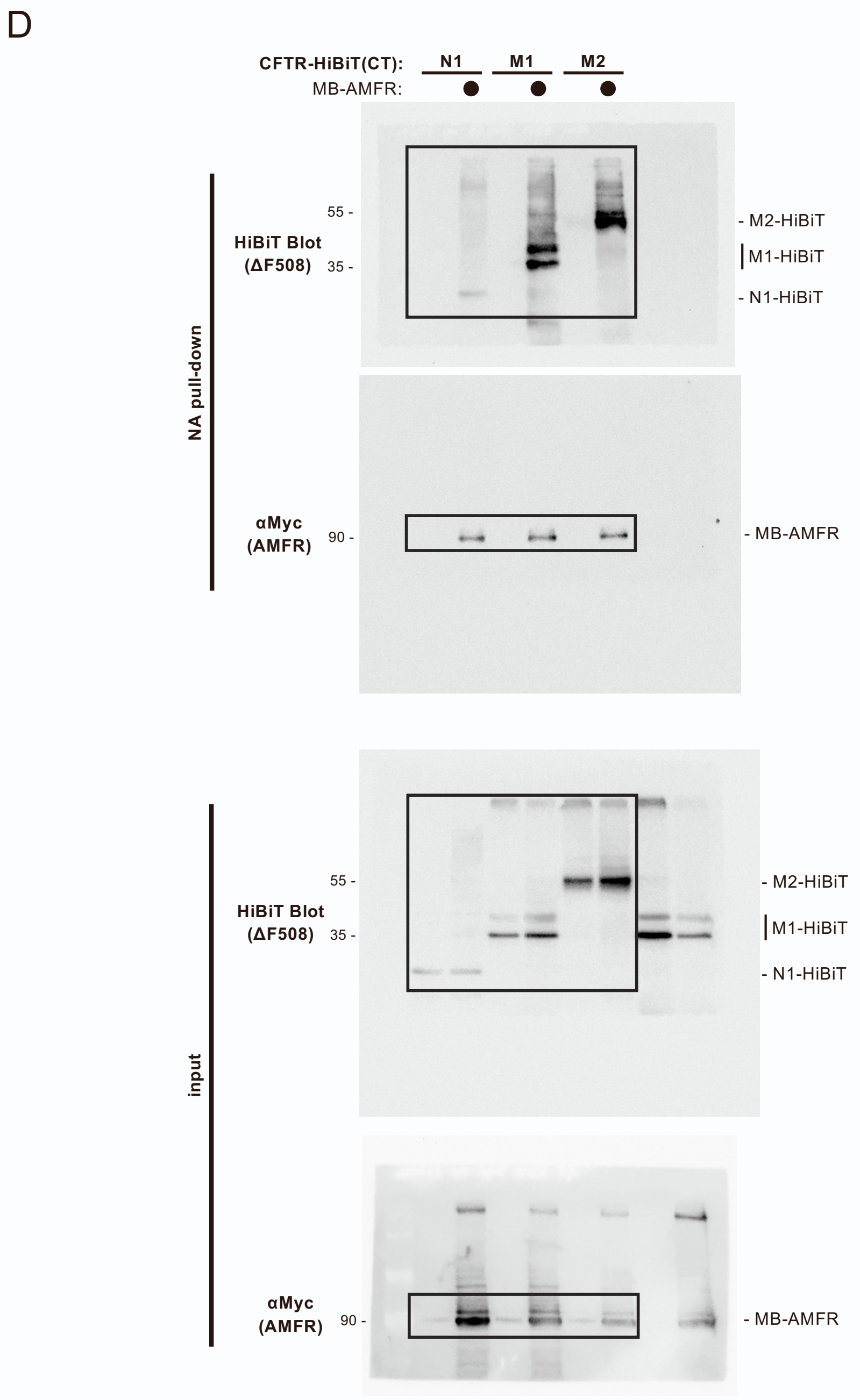
D
CFTR-HiBiT(CT):
N1
M1
M2
MB-AMFR:
NA pull-down
HiBiT Blot
(ΔF508)
55 -
35 -
- M2-HiBiT
M1-HiBiT
- N1-HiBiT
αMyc
(AMFR)
90 -
- MB-AMFR
input
HiBiT Blot
(ΔF508)
55 -
35 -
- M2-HiBiT
M1-HiBiT
- N1-HiBiT
αMyc
(AMFR)
90 -
- MB-AMFR

Figure S2

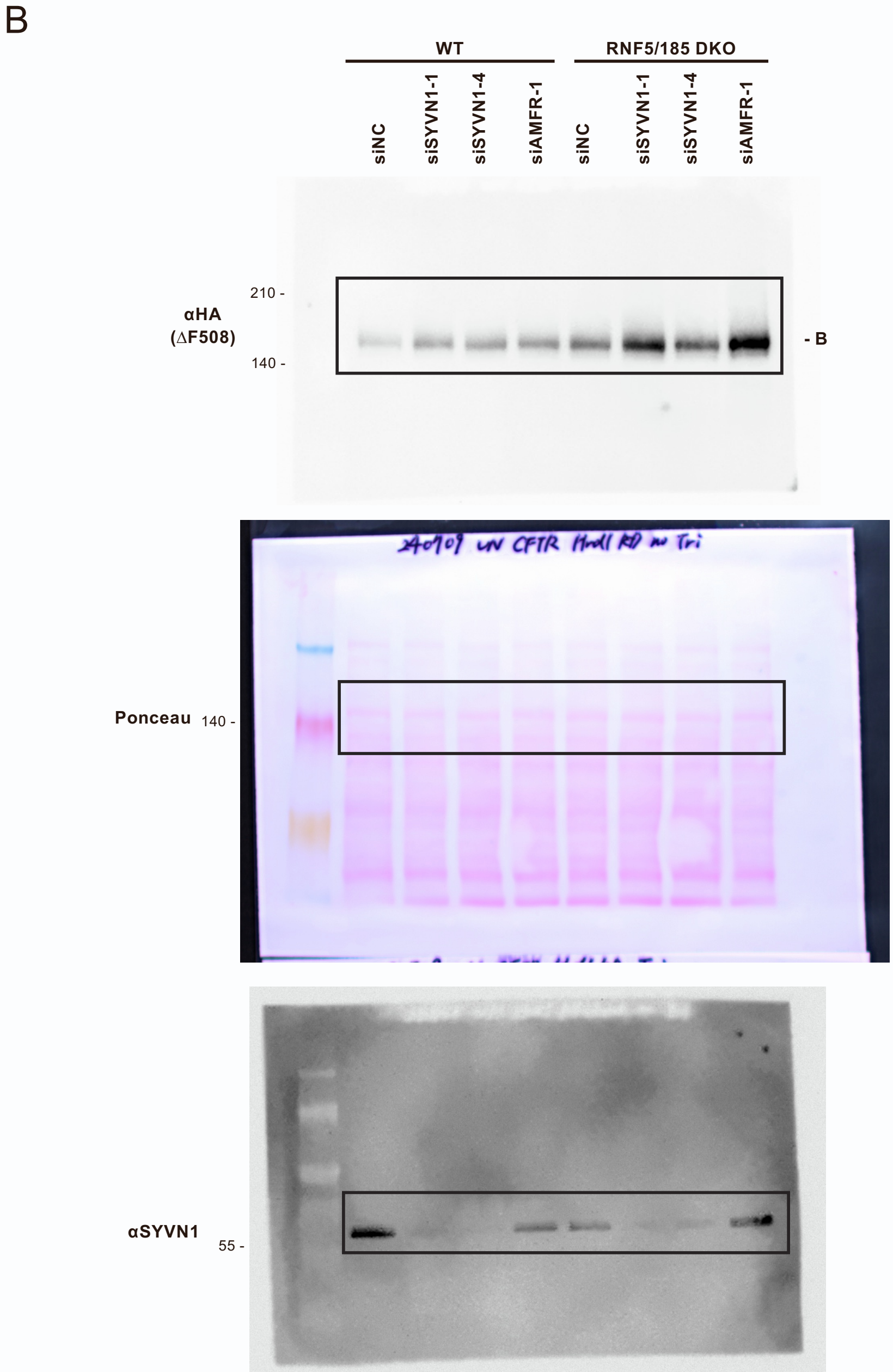
B
WT
RNF5/185 DKO
siNC
siSYVN1-1
siSYVN1-4
siAMFR-1
siNC
siSYVN1-1
siSYVN1-4
siAMFR-1
αHA
(ΔF508)
210 -
140 -
- B
Ponceau
140 -
αSYVN1
55 -
